# Insights into Cardiomyocyte Regeneration from Screening and Transcriptomics Approaches

**DOI:** 10.3390/ijms27020601

**Published:** 2026-01-07

**Authors:** Daniela T. Fuller, Aaron H. Wasserman, Ruya Liu

**Affiliations:** 1 Department of Medicine, University of Maryland School of Medicine, Baltimore, MD 21201, USA; daniela.fuller@som.umaryland.edu; 2 Department of Medicine, Michigan State University College of Human Medicine, East Lansing, MI 48824, USA; awasserm@msu.edu

**Keywords:** cardiac regeneration, high-throughput screening, cell heterogeneity, dedifferentiation, proliferation

## Abstract

Human adult cardiomyocytes (CMs) have limited regenerative capacity, posing a significant challenge in restoring cardiac function following substantial CM loss due to an acute ischemic event or chronic hemodynamic overload. Nearly half of patients show no improvement in left ventricular ejection fraction during recovery from acute myocardial infarction. At baseline, both humans and mice exhibit low but continuous cell turnover originating from the existing CMs. Moreover, myocardial infarction can induce endogenous CM cell cycling. Consequently, research has focused on identifying drivers of CM rejuvenation and proliferation from pre-existing CMs. High-throughput screening has facilitated the discovery of novel pro-proliferative targets through small molecules, microRNAs, and pathway-specific interventions. More recently, omics-based approaches such as single-nucleus RNA sequencing and spatial transcriptomics have expanded our understanding of cardiac cellular heterogeneity. The big-data strategies provide critical insights into why only a subset of CMs re-enter the cell cycle while most remain quiescent. In this review, we compare several high-throughput screening strategies used to identify novel targets for CM proliferation. We also summarize the benefits and limitations of various screening models—including zebrafish embryos, rodent CMs, human induced pluripotent stem cell-derived cardiomyocytes (iPSC-CMs), and cardiac organoids—underscoring the importance of integrating multiple systems to uncover new regenerative mechanisms. Further work is needed to identify translatable and safe targets capable of inducing functional CM expansion in clinical settings. By integrating high-throughput screening findings with insights into CM heterogeneity, this review provides a comprehensive framework for advancing cardiac regeneration research and guiding future therapeutic development.

## 1. Introduction

The hearts of zebrafish, newt, and neonatal mice can fully grow back and achieve complete functional recovery even after 20% ventricular apical resection [1,2,3,4]; however, adult mammalian hearts respond to cardiomyocyte (CM) loss from ischemic or hemodynamic stress with fibrotic remodeling, diminished contractility, and progressive heart failure. During development, mammalian CMs undergo two DNA synthesis peaks—one in mid-embryonic and the other at early postnatal periods: the former accounts for the cell number growth, and the latter results in hypertrophic growth—before permanently exiting the cell cycle. In mice, the two peak DNA synthesis periods occur at embryonic day 12 and postnatal day 4–6 [5]. At the second peak, CMs go through a final cell division, in which karyokinesis happens yet not cytokinesis, thus halt as binucleated cells, which is known as “acytokinetic mitosis”. This leads to polyploidy in mammalian CMs, providing structural stability but limiting proliferative potential [6,7,8,9].

For decades, adult CMs were believed to be postmitotic, and regenerative efforts largely centered on identifying cardiac stem or progenitor cells; however, these attempts yielded conflicting and inconclusive results [10,11]. Later studies demonstrated low (<1%) but continuous CM turnover at baseline [12,13,14,15] and modest cell-cycle reentry (3%) following myocardial infarction (MI) [16,17,18] in both humans and mice, suggesting an intrinsic—though limited—capacity for regeneration. A recent study showed a >6-fold increase of CM renewal in heart failure patients receiving left ventricle support device therapy relative to the healthy heart, further corroborating a substantial CM regeneration potential in diseased human hearts [19]. We learned from zebrafish and adult mouse studies that the regenerated CMs arise primarily from existing cells that transiently dedifferentiate, undergo metabolic reprogramming from oxidative phosphorylation to glycolysis, and attempt cytokinesis [1,20,21,22,23,24]. Only a small fraction complete true division in adult mouse hearts [20], highlighting the trade-off between proliferation and maturation [25,26,27]. Therefore, targeting CM proliferation programs to generate functional CM expansion is a promising strategy to promote heart regeneration.

High-throughput screening (HTS) approaches have been widely used to screen compounds or molecular targets that can induce CM proliferation, usually by using cell-cycle activity as a functional phenotypic readout. There are other screening strategies targeting predefined molecular targets that are known to induce CM proliferation. While labor-intensive, these unbiased methods have revealed novel regulators of CM proliferation and provided valuable insights into CM biology. Further, recent advances in single-cell and spatial transcriptomics uncovered marked heterogeneity among CMs, elucidating the distinct transcriptional or epigenetic signatures between the rare cycling population and the quiescent majorities. Integrating phenotypic screening with omics-based analyses of CM heterogeneity offers a powerful framework for identifying endogenous drivers of CM proliferation at baseline and in heart diseases.

A key challenge in developing therapeutics targeting cardiac regeneration lies in finding targets that can safely stimulate CM proliferation without impairing cardiac function or causing off-target effects. This is when integrating a three-dimensional heart organoid HTS model could be helpful in profiling the contractile and dilatation features before proceeding to the in vivo models. Together, these complementary approaches may accelerate the discovery of translatable targets and advance the development of effective regenerative therapies for cardiac repair.

## 2. Screening Models

### 2.1. Two-Dimensional Cultured Cells

Two commonly used two-dimensional (2D) models for screening compounds and targets that induce CM proliferation are murine neonatal cardiomyocytes (NCMs) and human induced pluripotent stem cell derived CMs (iPSC-CMs). NCMs are considered more mature than iPSC-CMs; however, iPSC-CMs offer the potential advantage of improved translatability due to their human genetic background. Despite this, iPSC-CMs remain immature, as they transcriptionally and functionally resemble fetal cardiomyocytes (see review [28]). Additional differences between human and murine CMs include heart rate, ion channel expression, and metabolic profiles [29]. Moreover, human CMs have a higher proportion of mono-nucleation and polyploidy than mouse or rat CMs [8,30,31]. Collectively, these species- and model-specific differences may contribute to variability in responses during regenerative therapeutic screening and should be considered when interpreting comparative results.

Another potential 2D screening approach involves the isolation of adult rat or mouse CMs. Given the limited availability of healthy adult human CMs, this strategy may be advantageous when a more mature CM model is desired. Traditionally, adult CMs are isolated using Langendorff perfusion; however, alternative methods have been developed, including aortic clamping with antegrade perfusion of the heart [32].

NCMs are usually isolated from 0–3-day-old neonatal rat or mouse heart ventricles [33,34,35,36]. They can be obtained in large numbers using relatively simple enzymatic digestion protocols (approximately 4 × 10^6^ CMs per rat heart and 1.5 × 10^6^ CMs per mouse heart), at low cost. Although NCMs are amenable to genetic modification post-isolation, plasmid uptake is inefficient, making standard transfection challenging. Viral transduction methods, including adenoviral and lentiviral vectors, as well as small interfering RNA (siRNA)-mediated knockdown, remain the most efficient approaches for genetic manipulation of NCMs. For example, Magadum et al. [37] used adenoviral delivery of a fluorescent ubiquitination-based cell cycle indicator (FUCCI) system [38], which labels G0-G1 phase cells with a Cdt1 marker and S-G2-M phase cells with a Geminin marker, to screen a compound library of 74 nuclear receptor ligands and 54 epigenetic modifiers at three concentrations in NCMs from 3-day-old rats. Eight compounds increased the proportion of Geminin-positive CMs, with carbacyclin, a PPARγ agonist, showing the strongest effect. Subsequent trans-genetic and MI models confirmed that PPARγ activation promotes proliferation in adult CMs. This study provided the first in vivo evidence that PPARγ is a bona fide regulation of CM proliferation, extending its established role in fatty acid oxidation and mitochondrial biogenesis.

In a large-scale screen of ~11,000 chemicals, Du and Zheng et al. implemented a high-content microscopy workflow in which FUCCI reporter NCMs from rats were used for primary screening, followed by confirmation of cytokinesis using mosaic analysis with double markers (MADM) reporters in mouse NCMs [39,40]. The MADM system uses two knock-in chimeric alleles encoding nonfunctional N- and C-terminal fragments of GFP and RFP (N-RFP/C-GFP and N-GFP/C-RFP). Cre-mediated inter-chromosomal recombination during S phase restores full-length GFP and/or RFP in daughter cells [41]. Coupling this genetic strategy with an interaction–prediction algorithm led to the discovery of a five-small-molecule (5SM) cocktail targeting α1 adrenergic receptor, JAK1, DYRKs, PTEN, MCT1, and linked these perturbations to lactate-LacRS2 signaling [39].

The advent of iPSC technology has made it possible to generate relatively pure populations of human CMs using small molecules and growth factors [42]. In one high-content screen, 5094 compounds were evaluated for their ability to induce DNA synthesis in human iPSC-CMs, as assessed by EdU (5-ethynyl-2-deoxyuridine) incorporation [43]. Six L-type calcium channel blockers ranked in the top 1% of positive hits, illustrating how phenotypic screens can support drug repurposing. Because CMs and non-CM cell types may respond differently across species and culture systems, combining rodent and human in vitro models is likely to be more informative than relying on a single model. For instance, Mohamed et al. performed transcriptomic profiling to identify 15 candidate cell-cycle regulators, then screened them in mouse NCMs. A cocktail of three regulators produced a ~200-fold increase in CM proliferation [44]; however, many cells died shortly after division. The authors therefore turned to a systematic screen of additional regulators in post-mitotic human iPSC-CMs (day 60) and discovered that combined expression of *cyclin B1*, *cyclin D1*, *CDK1*, and *CDK4* induced proliferation in ~20% of CMs. These studies demonstrate that 2D systems are powerful platforms for identifying candidate regulators; however, they lack the diverse mix of cardiac cell types and three-dimensional tissue architecture of the heart, and they do not provide functional readouts of syncytial contractility.

### 2.2. Three-Dimensional Human Cardiac Organoids

Since the first reports of three-dimensional (3D) in vitro cardiac constructs around 1999–2000 [45,46], substantial progress has been made in generating human cardiac organoids (hCOs) that recapitulate key features of heart development, architecture, and cell–cell interactions (see reviews by Lewis-Israeli et al. and Miyamoto et al. [47,48]). hCOs derived from human cells allow functional screening of heart rate, force generation, excitation and relaxation times, and arrhythmogenicity [49,50]. This is particularly important for CM proliferation screens, as candidate compounds must not only increase CM number but also preserve—or improve—cardiac function. hCOs are especially attractive considering the ethical and practical limitations of using large-scale in vivo mouse screening.

As organoid complexity continues to increase, hCOs are poised to become a preferred platform for simultaneous screening of CM proliferation and function. Mills et al. developed a hCO system that supports parallel assessment of iPSC-CM proliferation (Ki67 immunofluorescence), contractile force, and maturation status (MLC2v expression) [51]. In an initial study using this system, they screened different concentrations of glucose, palmitate, and insulin to identify a “maturation media” that enhanced force generation, increased metabolic and transcriptional maturity, and reduced proliferation. A subsequent screen of 105 compounds in the same platform identified nine mitogenic hits, two of which increased CM proliferation without impairing contractile function in mature hCOs [49]. Proteomics analysis implicated the mevalonate pathway (cholesterol biosynthesis) as the dominant target, and in vivo validation in adult mice confirmed pro-proliferative effects.

Organoid models that do not provide direct contractility readouts can still be valuable for toxicity testing and developmental studies. A major hurdle in hCO technology has been achieving sufficient vascularization to support larger, more complex, and better-perfused 3D structures. In a recent study [52], 34 small-molecule and growth factor combinations were screened to identify optimal differentiation conditions that generate multilineage, vascularized hCOs. These advanced models resemble human embryonic hearts at approximately 6.5 weeks post-conception. Notably, the same cocktail used to induce vascularization in the hCOs also induced blood vessel differentiation in hepatic organoids, suggesting conserved vasculature development across organs.

Kostina et al. recently generated human heart organoids (hHOs) that incorporate neural crest-derived tissues for the first time [53]. By allowing neurospheres to migrate into hHOs at the peak of SEMA3C expression, they established a system to study neural crest integration and used it to screen for neural crest abnormalities induced by commonly prescribed antidepressants. Similarly, O’Hern et al. developed assembloids that integrate tissue-resident macrophages into hHOs, enabling screens for pro-inflammatory factors that trigger arrhythmias [54]. Other recent advances in hCO technologies include incorporation of endoderm tissues to generate multi-lineage structures [55,56], blood-forming hCOs to model hematopoiesis [57], and ‘epicardioids’ to study non-CM cell types in greater detail [58]. These complex assembloids now support high-throughput and high-content screens for phenotypes beyond proliferation, including drug toxicity, electrophysiological changes, and developmental defects. Despite these advantages, organoids have limitations, including technical challenges in culture, high costs of functional instrumentation, and differences in tissue architecture compared with the in vivo heart [59].

### 2.3. Zebrafish Embryos

Zebrafish embryos have become a widely used in vivo screening model because they develop externally and rapidly, are transparent, and can be generated in large numbers from a single clutch. Numerous transgenic lines further expand their utility [60]. Phenotypic screens are commonly performed by placing embryos in multi-well plates, exposing them to compound libraries, and comparing treated embryos with controls for specific developmental or cellular phenotypes [61]. Readouts often include overt morphological features such as fin and tail structure, pigmentation, edema, and patterning defects (see review by Williams et al. [62]), cellular endpoints such as CM proliferation can also be quantified.

For proliferation assays, treatment around 3 days post-fertilization (dpf) is frequently used [63,64]. In a notable study, Han et al. applied the FUCCI system in zebrafish embryos to screen 1200 compounds for CM mitogenic effects [64]. They identified 104 compounds that increased and 26 that decreased CM proliferation, with many hits targeting steroid hormone pathways. Two Vitamin D analogs, alfacalcidiol and calcipotriene, increased the number of FUCCI-positive CMs by 141% and 100%, respectively. Follow-up experiments in both injured and uninjured adult zebrafish hearts showed that Vitamin D signaling regulates CM mitosis and influences heart and body size. Additional zebrafish screens have identified Wnt/β-catenin pathway inhibitors [65,66] and Hedgehog (Hh) and insulin-like growth factor (IGF) pathway agonists [63] as CM proliferation inducers.

Although zebrafish are powerful for large-scale in vivo screening, translation to mammals is less straightforward. Zebrafish hearts differ from mammalian hearts in genetic similarity, anatomical complexity, and CM ploidy. Adult zebrafish retain a high fraction of mononucleated, diploid CMs and maintain robust regenerative capacity, whereas mammalian CMs are frequently binucleated and polyploid [67]. Zebrafish models are therefore best used as initial discovery platform. Hits identified in embryos should be validated in mammalian systems, such as mouse, rat, or human cell-derived models, to confirm translatability [68]. When used in this complementary manner, zebrafish embryos are a valuable component of CM proliferation screening pipelines. Having outlined major in vitro and in vivo screening models, we next discuss common screening approaches and platforms that have been applied to these systems.

## 3. Screening Approaches and Platforms

### 3.1. In Vitro Screens

Common screening approaches for CM proliferation are summarized in Table 1. Small-molecule libraries remain the most frequently used tools, often deployed in 96-, 384-, or 1536-well plate formats to maximize throughput. A central challenge in phenotypic small-molecule screening is choosing appropriate concentration ranges. Compounds tested at excessively low doses may fail to show activity, whereas high doses increase the risk of toxicity and off-target effects. When optimal concentrations are unknown, testing across a log-scale range (e.g., 0.1, 1, and 10 µM) is recommended [43,49]. Concentrations above 10 µM are generally avoided for translational safety [69], and candidate drugs with IC_50_ values < 0.01 µM may raise safety concerns.

Some small-molecule screens focus on defined classes, such as kinase inhibitors [70,71,72,73], while others use broader collections that comprise thousands of chemically diverse compounds [39,43,64,74]. Approaches beyond small molecules include genetic or RNA-based perturbations: overexpression or knockdown of cell-cycle regulators [44], cardiac-enriched genes [75], microRNA (miRNA) mimics and anti-miRNAs [76], long non-coding RNAs (lncRNAs) [77], and extracellular factors [76,78]. More targeted screens have narrowed their focus to libraries of ginsenosides [79], glucocorticoid receptor agonists [80], or curated nuclear receptor and epigenetics regulator sets [37]. Together, these strategies reflect an active effort to identify CM proliferation modulators using both high-throughput and hypothesis-driven designs.

**Table 1:** Summary of CM proliferation screens, methods, and hit targets.

Screening Approach	Description	Examples/Notes	Hit Targets
**In silico screens**	Computational prediction of drug targets, miRNAs, or lncRNAs	Docking simulations, network modeling [81], public databases (HMDD for miRNAs, NONCODE for lncRNAs)	MEIS1 and Hoxb13 inhibition [82] lncRNA CPR [77] miRNA-1a/miRNA-15b combination [83]
**Gene/molecular expression screens**	Expression profiling to identify candidate genes or RNAs	Microarray, developmental gene comparisons, cardiogenesis-specific genes	*FoxM1* and *Id1* overexpression plus Jnk3 inhibition [84] *CDK1/CCNB/CDK4/CCND* combination [44] Tbx6 [75]
**miRNA/lncRNA library screens**	High-throughput screens of miRNA mimics, inhibitors, or siRNA against lncRNAs	hiPSC-CM miRNA screens with mimic libraries	96 active miRNA mimics [71] hsa-miR-590-3p and hsa-miR-199a-3p [85] miR-515-3p and miR-519e-3p [86]
**Extracellular factor screens**	Treatment with growth factors or ligands	Testing factors ± p38 inhibition	NRG1 [78] FGF1, IL-1, and NRG-1-β1 [76]
**Kinase inhibitor screens**	Libraries of small-molecule kinase inhibitors	Automated imaging-based quantification	Diarylamide and Diarylurea [70] Acadesine, Palomid-529, and SB216763 [72] BIO, SU1498, and KN93 [69]
**High-throughput chemical compound screens**	Large-scale screens of pharmaceutical libraries	FUCCI system or other imaging platforms	Nitrendipine and Verapamil [43] Phenylephrine Hydrochloride, Baricitinib, Harmine, Vo-ohpic Trihydrate, and AZD3965 [39] Carbacyclin [37] Osajin, Efaroxan Hydrochloride, Peruvoside, and Convallatoxin [87] Chicago Sky Blue [74]
**Zebrafish chemical screens**	In vivo phenotypic screens	Phenotypes	SAG, NBI-31772 [63] Alfacalcidol and Calcipotriene [64]
**Cardiac organoid screens or microbioreactor arrays**	Screens in cardiac organoids or high-density microfluidic array	Sometimes followed up by secondary screens in fibroblasts for CM selectivity [49]	Nifedipine, RRAD overexpression [50] CHIR99021 [88]
**Targeted screens**	Focused screens of plant-derived compounds, receptor families, or centrosome markers	Ginsenosides, FDA-approved GR agonists, centrosome-focused assays	20(R)-ginsenoside Rh2 [79] Mifepristone or GR KO [80] Nitrendipine and 1-NA-PP1 [89]

Abbreviations: CM: Cardiomyocyte; FUCCI: Fluorescent Ubiquitination-Based Cell Cycle Indicator; GR: Glucocorticoid Receptor; hiPSC-CM: Human Induced Pluripotent Stem Cell-Derived Cardiomyocyte; HMDD: Human MicroRNA Disease Database; KO: Knockout; lncRNA: Long Non-Coding RNA; miR/miRNA: Micro RNA; siRNA: Small Interfering RNA.

### 3.2. The Application of In Silico Pre-Screens

Pairing in silico analyses with in vitro and in vivo validation can increase screening efficiency and reduce library size. For example, Ponnusamy et al. mined microarray data available on NONCODE to identify lncRNAs with high expression in the mouse heart [77]. They then profiled these lncRNAs across postnatal and adult developmental stages and focused on candidates with differential expression patterns for functional screening.

Cheng et al. performed microarray analysis on isolated mouse CMs before and after two days of iPSC reprogramming, focusing on genes associated with mitosis [84]. This comparison between d0 and d2 reprogramming narrowed down the candidate list to nine differentially expressed genes. Another study used the Human microRNA Disease Database (HMDD) to identify miRNAs linked to MI and anti-proliferation [82]. Locked nucleic acid (LNA) anti-miR reagents targeting 17 miRNAs were screened, and depletion of miR-1a/15b emerged as a top hit for promoting CM proliferation.

Docking-based in silico screens have been used to prioritize small molecules targeting protein complexes of interest. In one study by Ahmed et al., FDA-approved drugs were screened in silico against the crystal structure of MEIS1–HOXB13 complex [82]. From 107 initial candidates, docking narrowed the list to nine, and subsequent validation identified paromomycin and neomycin as strong proliferation inducers. Complementary to these approaches, Harris et al. constructed a regulatory network model of CM proliferation based on existing literature and used it to predict outcomes of 93.6% of independent experiments [81]. This model suggested that *YAP*-driven proliferation is attenuated by cMyc or PI3K inhibition.

These examples illustrate how bioinformatic analysis, microarray or RNA-seq profiling, and network modeling can focus downstream wet-lab screening on a manageable number of candidates. While big-data analyses require specialized expertise and lack the full physiological context of in vitro and in vivo systems, when applied thoughtfully they can accelerate target discovery and reduce experimental cost. For further reading on in silico drug design, including computer aided drug design, we recommend this review by Sarma et al. [90].

### 3.3. Summary of Screening Approaches

The optimal screening approach for inducing CM proliferation depends on available resources, technical expertise, and the specific research question. High-throughput chemical compound screens encompass multiple subtypes, including kinase inhibitor libraries, zebrafish chemical screens, cardiac organoid screens, and other targeted or phenotypic assays. A key consideration when selecting a chemical compound screening strategy is whether to include compounds with unknown targets. Unbiased libraries maximize the chance of discovering unexpected regulators but create substantial challenges for downstream target deconvolution and mechanistic interpretation, often requiring extensive follow-up studies to identify molecular targets (see review [91]). In contrast, target-directed screening strategies, such as gene or molecular expression screens, extracellular factor screens, miRNA and lncRNA library screens, in silico approaches, and other targeted assays, offer clearer mechanistic insight by design, as they interrogate predefined pathways.

Cost and scalability also differ across platforms. Zebrafish and in silico screens scale efficiently and are well suited for large libraries, whereas organoid-based systems and adult CM models are more resource intensive and lower throughput. In practice, multiple screening approaches can be applied sequentially, as demonstrated by Ahmed et al. in Section 3.2, or used for validation across orthogonal models, for example, by progressing from 2D cultures to 3D platforms and ultimately in vivo injury models. Parallel screening across complementary platforms, such as zebrafish embryos and 2D in vitro CM models, remains relatively uncommon but may represent a powerful strategy to prioritize candidates with robust and conserved pro-proliferative effects.

## 4. Design and Validation of CM Proliferation Screens

### 4.1. Readout

Most phenotypic CM proliferation screens rely on cell-cycle markers as primary readouts. Commonly used indicators include synthetic thymidine analogs BrdU (5-bromo-2’-deoxyuridine) and EdU (5-ethynyl-2′-deoxyuridine), Ki-67, and phospho-histone H3 (pHH3). A major caveat is that adult CMs can express these markers during polyploidization or binucleation without completing cytokinesis. Therefore, it is essential to confirm that candidate treatments ultimately increase CM number rather than simply promoting DNA synthesis [92].

Several groups have used imaging-based methods to quantify CM number or CM nuclei over time [43,70,72,74,93]. Woo et al. imaged nuclei before and after drug treatment using Hoechst staining and developed software to distinguish polyploidization, multinucleation, and completed cytokinesis [43]. This allowed unbiased, automated quantification of net CM gain per well. Other studies have complemented initial proliferation screens with additional markers such as Aurora kinase B (AURKB) or Anillin [94,95]. AURKB localizes to the midzone and midbody during anaphase and telophase [94], and the positional symmetry of AURKB staining relative to daughter nuclei helps discriminate dividing from binucleating CMs [95,96]. One study quantified this pattern and termed symmetric AURKB staining “sAuB” as a hallmark of true CM division [97]. In vivo and 3D applications, however, often require further markers to reduce false positives. For example, Anillin normally concentrates at the cortex of the contractile ring during furrow constriction and cytokinesis, whereas diffuse Anillin staining during anaphase indicates contractile ring defects that lead to binucleation [94]. Similarly, correct localization of RhoA and IQGAP3, key regulators of actomyosin ring assembly, have been shown to reliably distinguish dividing from binucleating CMs [98]. Thus, combining early and late-stage cell-cycle markers with structural assessment of midbodies and cleavage furrow formation provides a robust strategy for measuring CM proliferation.

More recent work has explored “non-traditional” structural readouts. Meng et al. observed that centrosome reassembly and centriolar Pericentriolar Material 1 (PCM1) staining increased in proliferating iPSC-CMs, a pattern recapitulated by nitrendipine treatment, a hit from their proliferation screen [89]. These results are consistent with evidence that centrosome proteins relocate to the nuclear envelope around the perinatal stage, contributing to cell-cycle arrest and terminal CM differentiation [99,100]. This developmental “centrosome reduction” is linked with CM maturation, and its disruption has been associated with pediatric dilated cardiomyopathy [101]. Given the complexity of the centrosome, additional work is needed to define which structural features most reliably mark CM dedifferentiation and proliferative competence.

Because antibody-based quantification of CM proliferation can be costly and labor-intensive in high-throughput format, genetic reporters and live imaging systems have become attractive alternatives [92]. The FUCCI system, discussed above, labels live G0/G1 versus S/G2/M phase cells using Cdt1 and Geminin reporters [38], and has been adapted to zebrafish and rodent NCMs [39,63,64]. Partial FUCCI systems that express only the Geminin reporter have also been used in rat NCMs and mouse stem-cell lines [37,87]. These models enable real-time tracking of proliferation, avoid fixation artifacts, and can be driven by CM-specific promoters to obviate co-staining with CM markers such as cTnT. The MADM system and other mouse-specific reporters provide orthogonal lineage-tracing approaches based on recombination events that occur only during mitosis [41]. A widely used transgenic line expresses mCherry-H2B under an αMHC promoter and has been combined with eGFP-anillin to simultaneously visualize CM nuclei and late mitotic structures [70]. Finally, a recently developed lineage tracing approach is the ProTracer (‘Proliferation Tracer’) system [102,103]. ProTracer combines Cre-lox and Dre-rox recombination to permanently label Ki67- or CyclinA2-positive CMs with GFP, enabling lifelong lineage tracing of proliferating cells within specific mouse cell lineages at high spatial resolution. This system has been used to document CM proliferation in the heart after MI and in response to pressure overload [104]. Such in vivo reporters lay the foundation of quantifying CM proliferation in whole-animal contexts [105].

### 4.2. Validation Model

Most CM proliferation screens now incorporate in vivo validation in adult rodent models of myocardial injury. The adult mouse MI model remains the standard for testing regenerative therapies [37,39,44,50,74,77,78,79,80,83,84,85]. Larger animals such as pigs provide closer approximation to human cardiac physiology and heart size [82], but their use is limited by cost and logistics [106]. Ischemia–reperfusion (IR) models are also used; compared with permanent MI, IR produces smaller and more variable infarcts but more closely mimics the clinical course in acute MI patients undergoing reperfusion therapy (primary percutaneous coronary intervention) [107,108,109,110]. Most regenerative studies still rely on permanent left anterior descending artery (LAD) ligation, likely due to its reproducibility and larger infarct size [107,111], and comparatively few studies have directly contrasted the same therapy in MI versus IR settings [112].

Considering the high translational significance of preclinical injury models in adult mammals, it is important to note which CM proliferation targets have been validated in these settings. To date, potential therapies such as L-type calcium channel (LTCC) blockers have been systematically tested in iPSC-CMs [43,89], hCOs [50], and rodent MI models [50]. While validation of the Hippo pathway effector YAP was conducted in rodent MI models over a decade ago [113] and has extended to iPSC-CMs [71] and computational modeling [81], this potential therapy is yet to progress to larger mammals. Other Hippo pathway components, however, have been tested in porcine MI [114]. Additional CM proliferation targets with substantial preclinical evaluation include NRG1 in mouse MI [78], MEIS1/HOXB13 inhibitors in pig MI [82], cell-cycle regulators in mouse [44] and pig [115] MI, and miRNA cocktails across multiple injury settings [83,85,116,117]. By contrast, several promising candidates remain confined to 2D cell culture [87], zebrafish [64], or hCOs platforms [49], and would benefit from validation in more advanced mammalian models.

Key endpoints following MI or IR include left ventricular ejection fraction, fractional shortening, infarct/scar size, and CM proliferation indices in treated and control groups. Establishing a robust MI model requires surgical expertise, and post-MI histologic analysis is complicated by disruption of myocardial architecture, especially in the border zone, making it difficult to distinguish CMs from non-CMs. An RNAscope-based approach using intronic probes targeting *Tnnt2* (cTnT) transcripts has been proposed to more reliably identify CM nuclei across the cell cycle and in embryonic hearts [118]. This technique improves the specificity of CM identification in injured tissue.

In vitro surrogates for ischemic injury are also being developed. For example, one study used 1% O_2_ hypoxia in cardiac organoids after a proliferation screen to model ischemic conditions [83]. Contractility decreased under hypoxia and was rescued by treatment with a candidate compound, illustrating how organoids can link proliferation and function in a disease-relevant context. Another group performed a miRNA screen in cells transiently exposed to 0.5% O_2_ for 2 h [86] and found that top-hit miRNAs (miR-515-3p and miR-519e-3p) increased EdU and pHH3 labeling more strongly in hypoxia than normoxia. These results may be related to developmental differences in hypoxia responses; fetal CMs proliferate more under hypoxia, whereas neonatal CMs show reduced proliferation [119]. Mechanical injury has also been used as a surrogate for MI. A recent study showed that a simple scratch assay in vitro can induce a border zone-like CM transcriptomic state similar to that seen after MI in vivo [120]. Together, these models expand the repertoire of tools for validating hits from primary screens.

**Figure 1:**
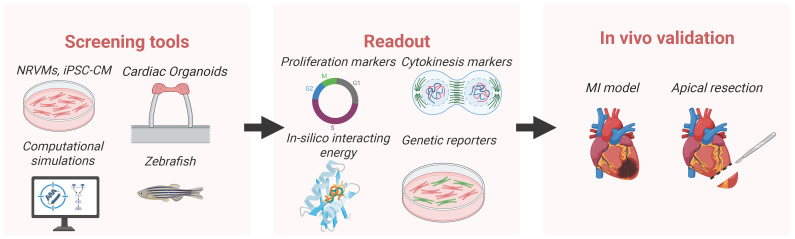
A typical pipeline for screening of CM proliferation therapeutics. Created in BioRender. Liu, R. (2026) Accessed on 24 December 2025, https://BioRender.com/8u9h63l.

Figure 1 summarizes a typical pipeline from initial screening to in vivo validation of CM proliferation therapeutics. Selected screens discussed in detail are also summarized in Table 1.

## 5. Insights into CM Heterogeneity from Single-Cell and Nucleus RNA Sequencing

### 5.1. Discovery of CM Proliferation Targets from Omics Data

Phenotypic screens provide unbiased access to novel mitogenic pathways but do not directly identify molecular targets. Omics technologies—particularly single-cell and single-nucleus RNA sequencing (scRNA-seq, snRNA-seq), spatial transcriptomics, and single-cell epigenomics—offer complementary tools to resolve CM heterogeneity and uncover proliferation-associated regulators. These methods can identify transcriptional differences between CM clusters, define proliferative subpopulations, and place them in anatomical context, such as border zone versus remote myocardium. In the following sections, we highlight studies that leverage omics approaches to define CM heterogeneity and identify proliferative CM subtypes and their regulatory targets.

### 5.2. Proliferative CM Subpopulations in the Embryonic and Postnatal Heart

**Figure 2:**
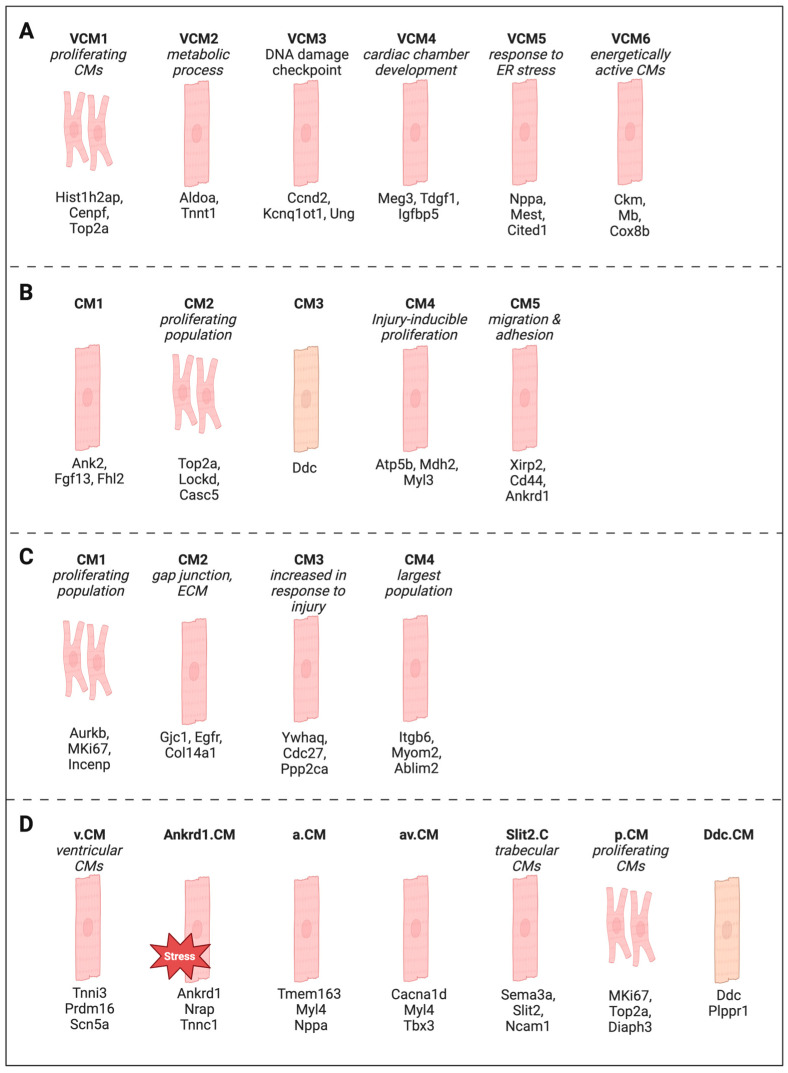
Selected publications featuring their identified CM subtypes along with several enriched or upregulated marker genes for each cluster. (**A**) Liu et al. data are from mice E8.5–E17.5 using scRNA-seq [121]. (**B**) Cui et al. data from mice 1D or 3D post-MI or sham surgery at P1 or P8 using snRNA-seq [122]. (**C**) Hao et al. data are from pig P1–P28 controls or apical resection at P1 using snRNA-seq [97]. (**D**) Wang et al. data are from mice P0–P21 using integrated spatial and snRNA-seq transcriptomics [123]. Created in BioRender. Liu, R. (2026) Accessed on 24 December 2025, https://BioRender.com/xbwmt0t.

Single-cell and single-nucleus sequencing have demonstrated that CMs are heterogeneous and can be subdivided into transcriptionally distinct populations (Figure 2). Liu et al. performed scRNA-seq on cardiac cells from seven embryonic timepoints (E8.5–E17.5) and identified six ventricular CM clusters (VCM1–VCM6) [121]. VCM1 exhibited the highest expression of cell-cycle genes and was classified as the proliferative CM population. Integrating VCM1-enriched genes with bulk RNA-seq co-expression networks led to the identification of three genes associated with loss of CM proliferative capacity during development. *PTMA* emerged as the top candidate, and its overexpression induced CM proliferation and improved repair after MI in adult hearts, illustrating how embryonic proliferative subpopulations can reveal targets relevant to adult regeneration.

Cui et al. compared regenerative (P1) and non-regenerative (P8) postnatal mouse hearts subjected to MI or sham surgery, with snRNA-seq performed at 1 and 3 days post-injury [122]. Five CM clusters were identified: CM1 (major population), CM2 (cell-cycle gene-enriched), CM3 (*Ddc* and a novel cDNA), CM4 (injury-responsive), and CM5 (injury-expanded). CM2 decreased modestly from 6% of CMs at P1 to 4% at P8, whereas CM4 declined with age but showed robust cell-cycle re-entry and activation of proliferation genes after injury. Although CM2 had a higher fraction of cells in G2/M at 3 days after P1 MI (~90% vs. ~70% in CM4), the larger size of CM4 (~28.5% vs. ~11.7% of CMs) made it the dominant proliferative population. CM4 was enriched in genes related to cell-cycle regulation, glycolysis, and ATP synthesis. Integrating snRNA-seq with scATAC-seq at 3 days post injury, the authors identified six candidate transcriptional regulators of CM4 based on motif accessibility and expression patterns. Co-expression of *NFYa* and *NFE2L1* in rat NCMs increased CM proliferation and conferred hypoxia resistance, and overexpression in non-regenerative hearts enhanced proliferation, improved ejection fraction, and reduced fibrosis after MI. This work illustrates the power of combining transcriptional and chromatin accessibility data to pinpoint regulators of proliferative CM populations.

In pig, Nakada et al. used apical resection at P1 followed by MI at P28 and performed snRNA-seq from P1 to P56 [124]. In this injury model [125,126], ~20% of the tip (apex) of the ventricle is physically removed, typically using scissors or a comparable tool, and the heart subsequently undergoes myocardial remodeling, fibrotic scar formation, and, depending on the age and species, varying degrees of regeneration. In this context, six CM populations were identified, and four showed increased proliferative probability. CMs subjected to early apical resection exhibited higher expression of S/G2/M-phase genes after MI than uninjured controls, suggesting that early injury may prime CMs with an epigenetic “memory” that facilitates later cell-cycle re-entry. A follow-up study by Hao et al. applied snRNA-seq to border zone and remote zone tissues after P1 apical resection [97]. Four CM subtypes were identified, including CM1, a proliferative cluster expressing mitosis and cytokinesis markers (*AURKB*, *MKI67*, *INCENP*, *CDCA8*, *BIRC5*), and CM3, which expressed genes related to centrosome and chromatin regulation (*CDC27*, *PPP2CA/B*, *AHCTF1*, *XPO1*) and showed *HIF1* activation. Overexpression of *HSPA5* or *HSP90B1* in a human CM line increased pHH3 labeling, linking stress-responsive pathways in CM3 to CM proliferation. These studies support a model in which specific neonatal CM subpopulations are primed to respond to injury with enhanced proliferative capacity, and their molecular signatures can be harnessed to design regenerative therapies.

### 5.3. CM Subpopulations in Mouse and Human Adult Heart

In adult mice (10–15 weeks), integrated scRNA-seq and snRNA-seq analyses indicate that approximately 0.4% of CMs are actively cycling, expressing *Ki67*, *Cenp*, *Kif23*, *Maf*, *C1qa*, *Lyz2*, *Sept7*, *Anln*, and *Aurkb* [127]. Following MI, Zhang et al. reported that 8.5% of a dedifferentiated CM subset expressed *Mki67* and cytokinesis genes (*Anln*, *Knl1*, *Kif11*), representing ~7.7% of total CMs [128]. Lineage tracing demonstrated that these proliferating CMs arise from pre-existing adult CMs rather than non-CM progenitors.

In human hearts, integrated scRNA-seq and snRNA-seq identified seven CM clusters in healthy and dilated cardiomyopathy samples [129]. Each cluster displayed distinct marker genes and pathway enrichment, including contraction, MAPK signaling, Notch signaling, and ion channel regulation, although proliferative signatures were not systematically assessed. Independent ^14^C dating studies confirmed that CM renewal occurs in adult human hearts, with Bayesian inference estimating an annual CM renewal rate of ~0.55% in healthy adults [19]. In ischemic and non-ischemic cardiomyopathy, renewal rates drop ~18- to 50-fold (0.01% and 0.03% per year, respectively), whereas patients with LVADs and improved LVEF (>5%) show ~5.6-fold higher renewal than healthy controls (3.1% per year). Disease state and mechanical unloading thus strongly modulate CM turnover in humans.

Once proliferation targets are identified, single-cell analyses of targeted models can provide mechanistic insight. For example, CM-specific overexpression of *YAP5SA* in adult mice induces Hippo pathway activation. Single-cell analysis showed that YAP5SA CMs transition through specific clusters associated with actin cytoskeleton remodeling, sarcomere disassembly, and G2/M-phase progression [130]. Pseudotime trajectories placed sarcomere disassembly early in the path to proliferation, followed by entry into G2/M-associated clusters, underscoring that structural remodeling is a prerequisite for adult CM cell-cycle re-entry.

### 5.4. Spatial Transcriptomics to Identify Border Zone CM Proliferation Programs

Spatial transcriptomics further refines our understanding of CM heterogeneity by preserving anatomical information. Border zone (BZ) CMs adjacent to the infarct core exhibit distinct transcriptional profiles compared with remote zone CMs, and these differences may be critical for proliferation. In zebrafish, BZ CMs undergo dedifferentiation and metabolic reprogramming toward glycolysis after injury, enabling cell-cycle re-entry, whereas remote CMs remain largely quiescent [24]. In adult mice, integrated single-cell and spatial analyses have revealed two spatially distinct BZ regions and shown that mechanical injuries (e.g., needle pass) recapitulate BZ-like expression patterns observed after MI [120].

One study used TOMO-seq, a spatial transcriptomics method based on sequentially sectioning hearts and performing bulk RNA-seq on 100 μm sections spanning ischemic, BZ, and remote zones [131]. Cross-species comparison of zebrafish and mouse BZ gene expression identified genes upregulated in the zebrafish BZ but not in the mouse. Prioritizing genes with regulatory functions and BZ enrichment led to the identification of three candidate proliferation targets, of which *Hmga1a* proved essential for zebrafish cardiac regeneration and non-lethal when knocked out. Overexpression of *Hmga1a* in adult mouse hearts promoted CM proliferation and improved post-MI function. TOMO-seq thus provides a spatially resolved platform to identify BZ-specific regulators that would be diluted in conventional bulk datasets.

### 5.5. Summary of CM Subpopulations

Across species and developmental stages, omics studies consistently show that CMs comprise one dominant population plus several smaller subpopulations with distinct transcriptional identities. In neonatal mouse and pig hearts, proliferating CMs represent a minor fraction of total CMs but expand markedly in response to injury [97,122]. Analyses of these proliferative and BZ-associated subpopulations have led to the discovery of regulators (e.g., *PTMA*, *NFYa/NFE2L1*, *Hmga1a*) that can promote proliferation in non-regenerative contexts.

In adult hearts, evidence indicates that a rare subset of CMs retains proliferative capacity. Omics-based characterization of these cells is still limited but suggests that sarcomere disassembly, metabolic remodeling, and specific transcriptional programs are required to permit re-entry into the cell cycle. Systematic definition of the transcriptomic and epigenetic signatures of proliferative adult-CM subpopulations remains an important goal for the field. These transcriptional and spatial maps set the stage for single-cell epigenetic analyses, which can define how chromatin accessibility constrains or enables these proliferation programs.

## 6. Non-CM Contributions to CM Proliferation

While Section 5 focused on heterogeneity within the CM compartment, omics approaches have also illuminated how non-CM populations sculpt the regenerative response. Inducing CM proliferation alone is unlikely to restore function without considering the surrounding microenvironment. Non-CM populations—including endothelial cells, immune cells, fibroblasts, and epicardial cells—shape extracellular matrix (ECM) composition, paracrine signaling, and angiogenesis, and thereby influence CM proliferation and regeneration. Here, we focus on insights from omics-based studies.

### 6.1. Spatial Transcriptomics and Non-CM Neighbors

Spatial transcriptomics provides a framework to identify non-CM populations that colocalize with proliferating CMs in the BZ. In several transgenic mouse models in which CM renewal is enhanced, proliferating CMs accumulate in BZ regions after MI [132,133,134]. Integrated single-cell and spatial analyses showed that BZ CMs are enriched for TEAD-binding motifs, Shroom3, conduction genes, and natriuretic peptides [120]. At the same time, specific non-CM clusters—type I interferon–expressing macrophages and neutrophils (IZ4), pro-inflammatory fibroblasts, and matricellular fibroblasts—were enriched in the BZ. Although their direct effects on CM proliferation were not fully delineated in that study, other work has started to define immune- and fibroblast-derived signals that regulate CM proliferation.

### 6.2. CM Maturation and Endothelial Cell Crosstalk

Haofei Wang et al. integrated snRNA-seq and spatial transcriptomics on mouse hearts from P0, P7, P14, and P21 and identified seven CM subclusters, including an *Ankrd1*-enriched stress-responsive cluster (Ankrd1.CM) [123]. Eleven spatial “neighborhoods” were defined based on co-localized cell types. Over time, a niche enriched in proliferative CMs (niche 9) declined, whereas a niche containing ventricular CMs and Ankrd1.CMs (niche 11) expanded. Capillary endothelial cell-rich niche 0 increased markedly during development.

Using a CM maturation index based on the expression of maturation-associated genes, the authors showed that the most mature CMs localized near capillary endothelial and pericyte populations. Cell–cell communication analysis identified *Ptprm-Ptprm* signaling between CMs and capillary endothelial cells from P7 onward, with *Meis1* as a downstream regulator promoting CM cell-cycle exit. Intrinsic gene regulatory network analysis highlighted 32 regulons involved in CM maturation, including *Thrb*, *Ppargc1a*, and *Esrra*. Machine-learning-based inference of extrinsic signaling revealed several ligand–receptor pairs (Nampt-Insr, Angpt2-Itga5, Angptl2-Itga5, Bmp5-Bmpr2, Igf1-Igf1r) likely to promote CM maturation. A high-throughput in vivo CRISPR screen (PIP-seq) targeting 50 predicted regulators identified *Rad21*, *Stat3*, and *Rreb1* as critical maturation genes. These data suggest that maturation is driven by both intrinsic programs and extrinsic endothelial signals. In principle, targeting such pathways might partially reverse CM maturation and restore proliferative competence.

### 6.3. Immune Cell Contributions

Single-cell analyses have revealed important roles for B cells, T cells, and macrophages in modulating CM proliferation. Tan et al. used single-cell sequencing and diphtheria toxin-mediated B cell ablation in adult mice to show that B cells promote CM proliferation after MI, potentially through secretion of Slpi, Ighg1, Lcn2, S100a8, S100a9, and Cxcl2 [135]. Li et al. found that FOXP3^+^ regulatory T cells (Tregs) promote neonatal CM proliferation via paracrine factors CCL24, GAS6, and AREG [136], and in vitro treatment of CMs with these factors increased Ki67 labeling. In parallel, Zhaoning Wang et al. identified macrophage-secreted AREG, CCL24, and CLCF1 as factors upregulated after P1 MI [137]; recombinant CCL24 increased rat NCM proliferation.

In zebrafish, single-cell analyses have defined multiple resident macrophage subpopulations that colocalize with BZ CMs and regulate ECM remodeling [138,139]. One macrophage cluster (mac3) expressed ECM-related genes, and BZ CMs were correspondingly enriched in ECM interaction pathways. Reanalysis of TOMO-seq data confirmed enrichment of *mmp14b* in the BZ, and *mmp14b* heterozygous mutants retained more scar tissue after injury without changes in CM proliferation, underscoring the importance of ECM remodeling for CM protrusion into the BZ. Collectively, these studies indicate that immune cells shape the structural and signaling environment required for regeneration.

### 6.4. Fibroblasts and ECM Signaling

Fibroblasts are central regulators of ECM composition and stiffness and exert profound effects on CM behavior. Following apical resection in P1 mice, Feng et al. used scRNA-seq to show that cardiac fibroblasts upregulate ECM genes [140]. Mass spectrometry of ECM components identified eight proteins as candidates for proliferation screening; versican emerged as a top hit based on Ki67 labeling. Versican injection after MI increased CM proliferation and improved cardiac function, demonstrating that ECM composition can be targeted to enhance regeneration.

In a separate study, Yin Wang et al. integrated single-cell datasets from P1–P56 mouse hearts and identified six fibroblast subtypes [141]. Fibroblast subtype switching during maturation was associated with changes in CM proliferation. Co-culture experiments showed that neonatal fibroblasts promoted higher CM proliferation (64% pHH3^+^ CMs) than adult fibroblasts (5% pHH3^+^). Pharmacologic inhibition of adult fibroblast-induced pathways with Plerixafor (chemokine signaling) or BP-1-102 (STAT3 inhibition) increased CM proliferation and improved function after MI. These results highlight fibroblasts as both drivers and potential therapeutic targets for CM proliferation.

### 6.5. The Epicardium and Epicardium-Derived Cells

Epicardium-derived progenitor cells (EPDCs) contribute fibroblasts and smooth muscle cells during development and after injury [142]. Whether they generate new CMs in mammals remains debated. In salamanders, single-cell and trajectory analyses showed that CLDN6^+^ EPDCs adopt CM-like transcriptional profiles after cryoinjury and express *Gata4*, *Gata6*, and *Myl3* [143,144], although the functional equivalence of these EPDC-derived cells to native CMs is not fully established.

In zebrafish, single-cell studies of injured and uninjured hearts have defined multiple epicardial subtypes, including clusters enriched in regenerating hearts [145]. One cluster shows high expression of proteoglycan link protein 1 (*hapln1*), which declines during mouse and human heart development. Zebrafish studies demonstrated that hapln1b is required for CM proliferation after injury via secretion of hyaluronic acid (HA). These findings suggest that epicardial-derived ECM components and signaling molecules may be harnessed to enhance mammalian heart regeneration.

### 6.6. Summary of Non-CM Contributions

Single-cell and spatial transcriptomics consistently show that non-CM populations are active participants in the regenerative response. Endothelial cells promote CM maturation through local signaling; B cells, Tregs, and macrophages secrete paracrine factors that enhance CM proliferation and ECM remodeling; fibroblasts regulate the ECM environment and CM proliferation through subtype switching; and epicardial cells contribute pro-regenerative signals and ECM components. These insights support the concept that targeting non-CM populations and their interactions with CMs may be as important as directly stimulating CM proliferation. (For more information on the contributions of non-CM populations to cardiac regeneration, we recommend this comprehensive review by Chen et al. [146]).

## 7. Single-Cell Epigenetics for Discovery of CM Proliferation Regulators

Single-cell ATAC-seq (scATAC-seq) profiles chromatin accessibility at the single-cell level and, when integrated with scRNA-seq, links regulatory elements to transcriptional output. Zhaoning Wang et al. performed scATAC-seq on neonatal mouse hearts 3 days after MI at P1 (regenerative) or P8 (non-regenerative) [137]. In CMs, P1 MI induced open chromatin regions enriched for PBX2 and SMAD motifs, whereas P8 MI induced regions enriched for FOSL1 and JUN motifs. Although CM subpopulations were not the main focus, distinct endothelial subtypes showed cluster-specific open chromatin at Gpihbp1 (vascular ECs), Npr3 (endocardial cells), and Fbln5 (arterial ECs). Further analyses focused on CM subsets are likely to clarify how epigenetic states constrain or permit proliferation.

Dong et al. applied scATAC-seq to zebrafish hearts at 0, 2, 7, and 14 days after apical resection [147]. Among non-CM populations, activated epicardial cells, fibroblasts, and endothelial cells showed open chromatin enriched for AP-1, Tead, and Stat motifs. Inhibition of AP-1 activity specifically in epicardial cells and fibroblasts impaired cardiac regeneration, demonstrating that AP-1-dependent programs in non-CMs are required for repair.

In fibroblast-to-iCM reprogramming, combined scRNA-seq and scATAC-seq across pseudotime revealed that Fos (AP-1 subunit) and Tcf21 act as barriers to CM induction [148]. Fos knockdown increased reprogramming efficiency, indicating that Fos-AP-1 activity can inhibit CM fate acquisition. Together, these studies show that single-cell epigenetic profiling is a powerful tool to identify transcription factor networks that either promote or restrict cardiac regeneration.

## 8. Safety Considerations and Clinical Translation

### 8.1. Biological and Translational Barriers to Cardiomyocyte Proliferation Therapies

One of the primary concerns associated with regenerative cardiac therapeutics is their potential oncogenic and off-target effects. Several strategies discussed in this review, including cyclin-dependent kinase/cyclin combinations, YAP activation, and pro-proliferative miRNA modulation, engage pathways that are also implicated oncogenic signaling. As a result, preventing unintended activation of proliferative programs in non-cardiac tissues represents a major translational challenge CM-based regenerative therapies.

Achieving effective and tissue-specific delivery is therefore critical for minimizing off-target risk. Multiple heart-targeted delivery approaches have been explored, including cardiotropic viral vectors, nanoparticle-based targeting strategies, carriers such as biodegradable polymers, and surgical or catheter-based delivery; for a comprehensive overview, we refer readers to the review by Sahoo et al. [149]. Among these strategies, intracoronary delivery techniques may offer advantages by enabling preferential targeting of the border zone, a region of the heart that is particularly responsive to proliferation cues following injury [150]. In addition, ultrasound-targeted microbubble destruction has been shown to enhance drug delivery to ischemic zones (see review [151]).

Beyond delivery specificity, incorporating mechanisms that allow temporal control and reversibility of proliferative signaling may be necessary to further enhance safety. Examples include genetic “off-switch” systems suitable for RNA-based regulation [152] and nanoparticle platforms incorporating inducible on–off switches (see review [153]). Together, the combination of heart-targeted delivery and built in safety control mechanisms represents a critical step toward reducing oncogenic risk and improving the translational feasibility of CM-proliferative therapies.

### 8.2. Risks of Chronic or Dysregulated Pro-Proliferative Signaling in the Adult Heart

Because sarcomere disassembly precedes CM proliferation, it must be followed by timely cell-cycle exit to permit CM redifferentiation and restoration of contractile function. Accordingly, therapeutic strategies aimed at inducing CM proliferation should enable transient and tightly controlled cell-cycle reactivation. One means of mitigating risk is precise temporal control over pathway activation, either through short treatment windows or through reversible systems that prevent sustained cell-cycle engagement and chronic proliferation.

In vivo testing of putative therapeutics is therefore essential to evaluate the risk of heart failure maladaptive cardiac remodeling. Such studies allow simultaneous assessment of CM proliferation and global cardiac function, including parameters such as ejection fraction. For example, induction of cell-cycle activity through increased cyclin D2 expression improved cardiac function in a model with increased afterload (transverse aortic constriction) but not in a model with increased preload (AV-shunt) [154]. Additionally, CM-specific deletion of Salvador (a Hippo pathway inhibitor) resulted in cardiomegaly, highlighting the potential consequences of dysregulated proliferative signaling [155]. Another major risk associated with CM dedifferentiation is arrhythmias due to electrical uncoupling in the setting of high levels of cell-cycle re-entry. Consistent with this concern, persistent expression of microRNA-199a in infarcted pig hearts led to fatal arrhythmias despite reductions in infarct size and improvements in cardiac function [116]. Collectively, these findings underscore the importance of transient and well-controlled activation of CM proliferation, coupled with rigorous in vivo validation of functional and electrophysiological outcomes.

### 8.3. Translational Progress and Regulatory Considerations

Once CM-proliferation candidates have been identified, extensive efforts are needed to advance them toward clinical trials. As discussed above, a critical step in establishing therapeutic potential is to demonstrate that interventions do not induce uncontrolled proliferation of CMs or other cell types and do not adversely affect cardiac function. Moreover, compounds that act on a single pathway may fail to produce functional benefits in advanced models, because cardiac regeneration in vivo is mediated by multiple intersecting signaling networks [156]. CM interactions with other cardiac cell types, such as fibroblasts, immune cells, and endothelial cells, must also be considered when validating screening hits [157]. From a regulatory perspective, these issues translate into stringent preclinical requirements for durability of benefit, off-target effects, and long-term safety, including arrhythmogenic and oncogenic risk.

Although no single platform fully captures the complexity of human heart disease, many of these challenges can be mitigated by performing staged, robust validation across complementary systems. These include 3D in vitro models to recapitulate multicellular composition and tissue-level function, large-animal injury models that approximate human cardiac size and physiology, and in silico or systems biology approaches that identify rational combinations and network-level effects [156].

Despite the inherent barriers, recent years have seen notable progress in advancing CM proliferation strategies into advanced preclinical trials. Prominent examples include microRNA-199a [116,158], cyclin D2 [115], *Salvador* knockdown [114], and CHIR99021-FGF1 nanoparticles [159], all of which have been evaluated in porcine injury models at minimum. While formal Phase I/II clinical trials of direct CM mitogens remain in development, one instructive example comes from congenital heart disease (CHD). In infants with tetralogy of Fallot with pulmonary stenosis, reduced CM cytokinesis was observed, and treatment with beta-blocker Propranolol increased CM proliferation and prevented adverse right ventricular remodeling in neonatal and adult injury models [160]. These findings supported initiation of an ongoing Phase I clinical trial assessing early-life propranolol therapy in 40 patients, with the goal of altering the management paradigm for this and potentially other CHDs [161]. The progression from mechanistic insights to preclinical validation and early-phase clinical trial illustrates a feasible translational pathway to future CM-proliferation-based interventions.

## 9. Discussion

In this review, we have summarized major screening models, platforms, and readouts for identifying CM-proliferation targets, alongside emerging omics approaches that resolve CM heterogeneity and the contributions of non-CM cell types. Traditional phenotypic screens—small-molecule libraries, genetic and RNA-based perturbations, and in silico pre-screens—remain essential for discovering candidate regulators. Newer approaches, including spatial transcriptomics, border zone analyses, non-CM transcriptomics, and single-cell epigenetics, provide complementary insights into how specific subpopulations and microenvironments support or restrict CM proliferation.

It is now broadly accepted that adult mammalian hearts lack progenitor- or stem cell-like populations that contribute meaningfully to CM regeneration. Instead, single-cell and single-nucleus analyses have revealed substantial heterogeneity within the CM compartment, with only a small fraction of cells expressing proliferation-associated gene signatures. Some subpopulations are poised to dedifferentiate and re-enter the cell cycle after injury, and others may retain epigenetic “memory” of prior damage that enhances proliferative responses. As noted above, the majority of adult human CMs are polyploid, creating a barrier to regeneration, whereas most adult zebrafish CMs are diploid and retain robust regenerative potential. It is likely that the rare human CMs that proliferate after injury are transcriptomically and epigenetically more similar to CMs from zebrafish and other regenerating models. Clarifying why only a minority of adult CMs can re-enter the cell cycle, while most remain quiescent, remains a central question that will require detailed single-cell comparisons between regenerating and non-regenerating organisms.

Non-CM populations also exert major control over CM proliferation and regeneration through ECM remodeling, paracrine signaling, and angiogenesis. Future regenerative strategies will likely require combination approaches that couple direct CM mitogenic stimuli with modulation of the surrounding niche. Across multiple models, pathways such as Hippo/YAP signaling, metabolic reprograming, and mevalonate/cholesterol biosynthesis have repeatedly shown pro-proliferative effects, whereas other candidate pathways remain preliminary and require further validation. Systematic integration of phenotypic screens with single-cell and spatial transcriptomics and epigenetic datasets will be essential to distinguish broadly relevant, convergent targets from model-specific findings, refine target selection, and prioritize those interventions most likely to be translatable. Ultimately, candidate targets must demonstrate durable functional benefit in clinically relevant injury models, be translatable to human systems, and meet stringent safety criteria. Avoiding oncogenic transformation and off-target toxicity represents one of the main translational barriers for CM-proliferation-based therapies. Ultimately, systematic integration of phenotypic screens with single-cell and spatial transcriptomic and epigenetic datasets will be required to triage candidate pathways, refine target selection, and focus translational efforts on those interventions most likely to be safe and effective in patients.

## Data Availability

No new data were created or analyzed in this study. Data sharing is not applicable to this article.

## Abbreviations

The following abbreviations are used in this manuscript:

2D	two-dimensional
3D	three-dimensional
AP-1	activator protein-1
AURKB	Aurora kinase B
BrdU	5-bromo-2′-deoxyuridine
BZ	border zone
CDK	cyclin-dependent kinase
CHD	congenital heart disease
CMs	cardiomyocytes
DNA	deoxyribonucleic acid
dpf	days post-fertilization
EC	endothelial cell
ECM	extracellular matrix
EdU	5-ethynyl-2′-deoxyuridine
EPDC	epicardium-derived progenitor cell
FGF	fibroblast growth factor
FUCCI	fluorescent ubiquitination-based cell cycle indicator
GFP	green fluorescent protein
HA	hyaluronic acid
Hh	Hedgehog
hiPSC	human induced pluripotent stem cell
hiPSC-CM	human induced pluripotent stem cell-derived cardiomyocyte
HTS	High-throughput screening
IGF	insulin-like growth factor
iCM	induced cardiomyocyte
IR	ischemia–reperfusion
iPSC	induced pluripotent stem cell
iPSC-CM	induced pluripotent stem cell-derived cardiomyocyte
LAD	left anterior descending (coronary artery) artery
lncRNA	long non-coding RNA
LNA	locked nucleic acid
LTCC	L-type calcium channel
LVAD	left ventricular assist device
LVEF	left ventricular ejection fraction
MADM	mosaic analysis with double markers
MAPK	mitogen-activated protein kinase
MI	myocardial infarction
miRNA	microRNA
NCM	neonatal cardiomyocyte
PCM1	Pericentriolar Material 1
pHH3	phospho-histone H3
PPAR	peroxisome proliferator-activated receptor
RNA-seq	RNA sequencing
S5M	five-small-molecule (cocktail)
scATAC-seq	single-cell assay for transposase-accessible chromatin sequencing
scRNA-seq	single-cell RNA sequencing
siRNA	small interfering RNA
snRNA-seq	single-nucleus RNA sequencing
TOMO-seq	spatially resolved RNA sequencing
Treg	regulatory T cell
YAP	Yes-associated protein
hCO	human cardiac organoid
hHO	human heart organoid

## References

[1] Poss K.D., Wilson L.G., Keating M.T. (2002). Heart regeneration in zebrafish. Science.

[2] Simon H.G., Odelberg S. (2015). Assessing cardiomyocyte proliferative capacity in the newt heart and primary culture. Salamanders in Regeneration Research: Methods and Protocols; Methods in Molecular Biology.

[3] Porrello E.R., Mahmoud A.I., Simpson E., Hill J.A., Richardson J.A., Olson E.N., Sadek H.A. (2011). Transient regenerative potential of the neonatal mouse heart. Science.

[4] Natarajan N., Abbas Y., Bryant D.M., Gonzalez-Rosa J.M., Sharpe M., Uygur A., Cocco-Delgado L.H., Ho N.N., Gerard N.P., Gerard C.J. (2018). Complement Receptor C5aR1 Plays an Evolutionarily Conserved Role in Successful Cardiac Regeneration. Circulation.

[5] Soonpaa M.H., Kim K.K., Pajak L., Franklin M., Field L.J. (1996). Cardiomyocyte DNA synthesis and binucleation during murine development. Am. J. Physiol..

[6] Orr-Weaver T.L. (2015). When bigger is better: The role of polyploidy in organogenesis. Trends Genet..

[7] Patterson M., Swift S.K. (2019). Residual Diploidy in Polyploid Tissues: A Cellular State with Enhanced Proliferative Capacity for Tissue Regeneration?. Stem Cells Dev..

[8] Patterson M., Barske L., Van Handel B., Rau C.D., Gan P., Sharma A., Parikh S., Denholtz M., Huang Y., Yamaguchi Y. (2017). Frequency of mononuclear diploid cardiomyocytes underlies natural variation in heart regeneration. Nat. Genet..

[9] Gan P., Patterson M., Sucov H.M. (2020). Cardiomyocyte Polyploidy and Implications for Heart Regeneration. Annu. Rev. Physiol..

[10] He L., Nguyen N.B., Ardehali R., Zhou B. (2020). Heart Regeneration by Endogenous Stem Cells and Cardiomyocyte Proliferation: Controversy, Fallacy, and Progress. Circulation.

[11] Doppler S.A., Deutsch M.A., Lange R., Krane M. (2015). Direct Reprogramming-The Future of Cardiac Regeneration?. Int. J. Mol. Sci..

[12] Bergmann O., Bhardwaj R.D., Bernard S., Zdunek S., Barnabe-Heider F., Walsh S., Zupicich J., Alkass K., Buchholz B.A., Druid H. (2009). Evidence for cardiomyocyte renewal in humans. Science.

[13] Senyo S.E., Steinhauser M.L., Pizzimenti C.L., Yang V.K., Cai L., Wang M., Wu T.D., Guerquin-Kern J.L., Lechene C.P., Lee R.T. (2013). Mammalian heart renewal by pre-existing cardiomyocytes. Nature.

[14] Ali S.R., Hippenmeyer S., Saadat L.V., Luo L., Weissman I.L., Ardehali R. (2014). Existing cardiomyocytes generate cardiomyocytes at a low rate after birth in mice. Proc. Natl. Acad. Sci. USA.

[15] Bergmann O., Zdunek S., Felker A., Salehpour M., Alkass K., Bernard S., Sjostrom S.L., Szewczykowska M., Jackowska T., dos Remedios C. (2015). Dynamics of Cell Generation and Turnover in the Human Heart. Cell.

[16] Bradley L.A., Young A., Li H., Billcheck H.O., Wolf M.J. (2021). Loss of Endogenously Cycling Adult Cardiomyocytes Worsens Myocardial Function. Circ. Res..

[17] Beltrami A.P., Urbanek K., Kajstura J., Yan S.M., Finato N., Bussani R., Nadal-Ginard B., Silvestri F., Leri A., Beltrami C.A. (2001). Evidence that human cardiac myocytes divide after myocardial infarction. N. Engl. J. Med..

[18] Malliaras K., Zhang Y., Seinfeld J., Galang G., Tseliou E., Cheng K., Sun B., Aminzadeh M., Marban E. (2013). Cardiomyocyte proliferation and progenitor cell recruitment underlie therapeutic regeneration after myocardial infarction in the adult mouse heart. EMBO Mol. Med..

[19] Derks W., Rode J., Collin S., Rost F., Heinke P., Hariharan A., Pickel L., Simonova I., Lazar E., Graham E. (2025). A Latent Cardiomyocyte Regeneration Potential in Human Heart Disease. Circulation.

[20] Wang W.E., Li L., Xia X., Fu W., Liao Q., Lan C., Yang D., Chen H., Yue R., Zeng C. (2017). Dedifferentiation, Proliferation, and Redifferentiation of Adult Mammalian Cardiomyocytes After Ischemic Injury. Circulation.

[21] Kikuchi K., Holdway J.E., Werdich A.A., Anderson R.M., Fang Y., Egnaczyk G.F., Evans T., MacRae C.A., Stainier D.Y., Poss K.D. (2010). Primary contribution to zebrafish heart regeneration by gata4(+) cardiomyocytes. Nature.

[22] Fajardo V.M., Feng I., Chen B.Y., Perez-Ramirez C.A., Shi B., Clark P., Tian R., Lien C.L., Pellegrini M., Christofk H. (2021). GLUT1 overexpression enhances glucose metabolism and promotes neonatal heart regeneration. Sci. Rep..

[23] Cardoso A.C., Lam N.T., Savla J.J., Nakada Y., Pereira A.H.M., Elnwasany A., Menendez-Montes I., Ensley E.L., Petric U.B., Sharma G. (2020). Mitochondrial Substrate Utilization Regulates Cardiomyocyte Cell Cycle Progression. Nat. Metab..

[24] Honkoop H., de Bakker D.E., Aharonov A., Kruse F., Shakked A., Nguyen P.D., de Heus C., Garric L., Muraro M.J., Shoffner A. (2019). Single-cell analysis uncovers that metabolic reprogramming by ErbB2 signaling is essential for cardiomyocyte proliferation in the regenerating heart. eLife.

[25] Guo Y., Pu W.T. (2020). Cardiomyocyte Maturation: New Phase in Development. Circ. Res..

[26] Zhao M.T., Ye S., Su J., Garg V. (2020). Cardiomyocyte Proliferation and Maturation: Two Sides of the Same Coin for Heart Regeneration. Front. Cell Dev. Biol..

[27] Singh B.N., Yucel D., Garay B.I., Tolkacheva E.G., Kyba M., Perlingeiro R.C.R., van Berlo J.H., Ogle B.M. (2023). Proliferation and Maturation: Janus and the Art of Cardiac Tissue Engineering. Circ. Res..

[28] Wu P., Deng G., Sai X., Guo H., Huang H., Zhu P. (2021). Maturation strategies and limitations of induced pluripotent stem cell-derived cardiomyocytes. Biosci. Rep..

[29] Tanner M.R., Beeton C. (2018). Differences in ion channel phenotype and function between humans and animal models. Front. Biosci. (Landmark Ed.).

[30] Wohlschlaeger J., Levkau B., Brockhoff G., Schmitz K.J., von Winterfeld M., Takeda A., Takeda N., Stypmann J., Vahlhaus C., Schmid C. (2010). Hemodynamic support by left ventricular assist devices reduces cardiomyocyte DNA content in the failing human heart. Circulation.

[31] Derks W., Bergmann O. (2020). Polyploidy in Cardiomyocytes: Roadblock to Heart Regeneration?. Circ. Res..

[32] Omatsu-Kanbe M., Fukunaga R., Mi X., Matsuura H. (2021). An Antegrade Perfusion Method for Cardiomyocyte Isolation from Mice. J. Vis. Exp..

[33] Pereira A.H.M., Cardoso A.C., Franchini K.G. (2021). Isolation, culture, and immunostaining of neonatal rat ventricular myocytes. STAR Protoc..

[34] Vandergriff A.C., Hensley M.T., Cheng K. (2015). Isolation and cryopreservation of neonatal rat cardiomyocytes. J. Vis. Exp..

[35] Bongiovanni C., Miano C., Sacchi F., Da Pra S., Del Bono I., Boriati S., D’Uva G. (2024). Protocol for isolating and culturing neonatal murine cardiomyocytes. STAR Protoc..

[36] Ehler E., Moore-Morris T., Lange S. (2013). Isolation and culture of neonatal mouse cardiomyocytes. J. Vis. Exp..

[37] Magadum A., Ding Y., He L., Kim T., Vasudevarao M.D., Long Q., Yang K., Wickramasinghe N., Renikunta H.V., Dubois N. (2017). Live cell screening platform identifies PPARdelta as a regulator of cardiomyocyte proliferation and cardiac repair. Cell Res..

[38] Sakaue-Sawano A., Kurokawa H., Morimura T., Hanyu A., Hama H., Osawa H., Kashiwagi S., Fukami K., Miyata T., Miyoshi H. (2008). Visualizing spatiotemporal dynamics of multicellular cell-cycle progression. Cell.

[39] Du J., Zheng L., Gao P., Yang H., Yang W.J., Guo F., Liang R., Feng M., Wang Z., Zhang Z. (2022). A small-molecule cocktail promotes mammalian cardiomyocyte proliferation and heart regeneration. Cell Stem Cell.

[40] Zheng L., Wang Z., Du J., Zhu X., Xiong J.W. (2022). Protocol to identify small molecules promoting rat and mouse cardiomyocyte proliferation based on the FUCCI and MADM reporters. STAR Protoc..

[41] Zong H., Espinosa J.S., Su H.H., Muzumdar M.D., Luo L. (2005). Mosaic analysis with double markers in mice. Cell.

[42] Lyra-Leite D.M., Gutierrez-Gutierrez O., Wang M., Zhou Y., Cyganek L., Burridge P.W. (2022). A review of protocols for human iPSC culture, cardiac differentiation, subtype-specification, maturation, and direct reprogramming. STAR Protoc..

[43] Woo L.A., Tkachenko S., Ding M., Plowright A.T., Engkvist O., Andersson H., Drowley L., Barrett I., Firth M., Akerblad P. (2019). High-content phenotypic assay for proliferation of human iPSC-derived cardiomyocytes identifies L-type calcium channels as targets. J. Mol. Cell. Cardiol..

[44] Mohamed T.M.A., Ang Y.S., Radzinsky E., Zhou P., Huang Y., Elfenbein A., Foley A., Magnitsky S., Srivastava D. (2018). Regulation of Cell Cycle to Stimulate Adult Cardiomyocyte Proliferation and Cardiac Regeneration. Cell.

[45] Bursac N., Papadaki M., Cohen R.J., Schoen F.J., Eisenberg S.R., Carrier R., Vunjak-Novakovic G., Freed L.E. (1999). Cardiac muscle tissue engineering: Toward an in vitro model for electrophysiological studies. Am. J. Physiol..

[46] Zimmermann W.H., Fink C., Kralisch D., Remmers U., Weil J., Eschenhagen T. (2000). Three-dimensional engineered heart tissue from neonatal rat cardiac myocytes. Biotechnol. Bioeng..

[47] Lewis-Israeli Y.R., Wasserman A.H., Aguirre A. (2021). Heart Organoids and Engineered Heart Tissues: Novel Tools for Modeling Human Cardiac Biology and Disease. Biomolecules.

[48] Miyamoto M., Nam L., Kannan S., Kwon C. (2021). Heart organoids and tissue models for modeling development and disease. Semin. Cell Dev. Biol..

[49] Mills R.J., Parker B.L., Quaife-Ryan G.A., Voges H.K., Needham E.J., Bornot A., Ding M., Andersson H., Polla M., Elliott D.A. (2019). Drug Screening in Human PSC-Cardiac Organoids Identifies Pro-proliferative Compounds Acting via the Mevalonate Pathway. Cell Stem Cell.

[50] Devilee L.A.C., Salama A.B.M., Miller J.M., Reid J.D., Ou Q., Baraka N.M., Abou Farraj K., Jamal M., Nong Y., Rosengart T.K. (2025). Pharmacological or genetic inhibition of LTCC promotes cardiomyocyte proliferation through inhibition of calcineurin activity. npj Regen. Med..

[51] Mills R.J., Titmarsh D.M., Koenig X., Parker B.L., Ryall J.G., Quaife-Ryan G.A., Voges H.K., Hodson M.P., Ferguson C., Drowley L. (2017). Functional screening in human cardiac organoids reveals a metabolic mechanism for cardiomyocyte cell cycle arrest. Proc. Natl. Acad. Sci. USA.

[52] Abilez O.J., Yang H., Guan Y., Shen M., Yildirim Z., Zhuge Y., Venkateshappa R., Zhao S.R., Gomez A.H., El-Mokahal M. (2025). Gastruloids enable modeling of the earliest stages of human cardiac and hepatic vascularization. Science.

[53] Kostina A., Kiselev A., Huang A., Lankerd H., Caywood S., Jurado-Fernandez A., Volmert B., O’Hern C., Juhong A., Liu Y. (2024). Self-organizing human heart assembloids with autologous and developmentally relevant cardiac neural crest-derived tissues. bioRxiv.

[54] O’Hern C., Caywood S., Aminova S., Kiselev A., Volmert B., Cao W., Wang F., Dionise M., Sewavi M.L., Skoric M. (2025). Human heart-macrophage assembloids mimic immune-cardiac interactions and enable arrhythmia disease modeling. Cell Stem Cell.

[55] Miao Y., Pek N.M., Tan C., Jiang C., Yu Z., Iwasawa K., Shi M., Kechele D.O., Sundaram N., Pastrana-Gomez V. (2025). Co-development of mesoderm and endoderm enables organotypic vascularization in lung and gut organoids. Cell.

[56] Silva A.C., Matthys O.B., Joy D.A., Kauss M.A., Natarajan V., Lai M.H., Turaga D., Blair A.P., Alexanian M., Bruneau B.G. (2021). Co-emergence of cardiac and gut tissues promotes cardiomyocyte maturation within human iPSC-derived organoids. Cell Stem Cell.

[57] Dardano M., Kleemiß F., Kosanke M., Lang D., Wilson L., Franke A., Teske J., Shivaraj A., de la Roche J., Fischer M. (2024). Blood-generating heart-forming organoids recapitulate co-development of the human haematopoietic system and the embryonic heart. Nat. Cell Biol..

[58] Meier A.B., Zawada D., De Angelis M.T., Martens L.D., Santamaria G., Zengerle S., Nowak-Imialek M., Kornherr J., Zhang F., Tian Q. (2023). Epicardioid single-cell genomics uncovers principles of human epicardium biology in heart development and disease. Nat. Biotechnol..

[59] Andrews M.G., Kriegstein A.R. (2022). Challenges of Organoid Research. Annu. Rev. Neurosci..

[60] Staudt D., Stainier D. (2012). Uncovering the molecular and cellular mechanisms of heart development using the zebrafish. Annu. Rev. Genet..

[61] Tamplin O.J., White R.M., Jing L., Kaufman C.K., Lacadie S.A., Li P., Taylor A.M., Zon L.I. (2012). Small molecule screening in zebrafish: Swimming in potential drug therapies. Wiley Interdiscip. Rev. Dev. Biol..

[62] Williams C.H., Hong C.C. (2016). Zebrafish small molecule screens: Taking the phenotypic plunge. Comput. Struct. Biotechnol. J..

[63] Choi W.Y., Gemberling M., Wang J., Holdway J.E., Shen M.C., Karlstrom R.O., Poss K.D. (2013). In vivo monitoring of cardiomyocyte proliferation to identify chemical modifiers of heart regeneration. Development.

[64] Han Y., Chen A., Umansky K.B., Oonk K.A., Choi W.Y., Dickson A.L., Ou J., Cigliola V., Yifa O., Cao J. (2019). Vitamin D Stimulates Cardiomyocyte Proliferation and Controls Organ Size and Regeneration in Zebrafish. Dev. Cell.

[65] Ni T.T., Rellinger E.J., Mukherjee A., Xie S., Stephens L., Thorne C.A., Kim K., Hu J., Lee E., Marnett L. (2011). Discovering small molecules that promote cardiomyocyte generation by modulating Wnt signaling. Chem. Biol..

[66] Xie S., Fu W., Yu G., Hu X., Lai K.S., Peng X., Zhou Y., Zhu X., Christov P., Sawyer L. (2020). Discovering small molecules as Wnt inhibitors that promote heart regeneration and injury repair. J. Mol. Cell Biol..

[67] Gonzalez-Rosa J.M., Sharpe M., Field D., Soonpaa M.H., Field L.J., Burns C.E., Burns C.G. (2018). Myocardial Polyploidization Creates a Barrier to Heart Regeneration in Zebrafish. Dev. Cell.

[68] Beffagna G. (2019). Zebrafish as a Smart Model to Understand Regeneration After Heart Injury: How Fish Could Help Humans. Front. Cardiovasc. Med..

[69] Dixit V.A. (2019). A simple model to solve a complex drug toxicity problem. Toxicol. Res..

[70] Carrillo Garcia C., Becker C., Forster M., Lohmann S., Freitag P., Laufer S., Sievers S., Fleischmann B.K., Hesse M., Schade D. (2022). High-Throughput Screening Platform in Postnatal Heart Cells and Chemical Probe Toolbox to Assess Cardiomyocyte Proliferation. J. Med. Chem..

[71] Diez-Cunado M., Wei K., Bushway P.J., Maurya M.R., Perera R., Subramaniam S., Ruiz-Lozano P., Mercola M. (2018). miRNAs that Induce Human Cardiomyocyte Proliferation Converge on the Hippo Pathway. Cell Rep..

[72] Neininger A.C., Long J.H., Baillargeon S.M., Burnette D.T. (2019). A simple and flexible high-throughput method for the study of cardiomyocyte proliferation. Sci. Rep..

[73] Uosaki H., Magadum A., Seo K., Fukushima H., Takeuchi A., Nakagawa Y., Moyes K.W., Narazaki G., Kuwahara K., Laflamme M. (2013). Identification of chemicals inducing cardiomyocyte proliferation in developmental stage-specific manner with pluripotent stem cells. Circ. Cardiovasc. Genet..

[74] Yifa O., Weisinger K., Bassat E., Li H., Kain D., Barr H., Kozer N., Genzelinakh A., Rajchman D., Eigler T. (2019). The small molecule Chicago Sky Blue promotes heart repair following myocardial infarction in mice. JCI Insight.

[75] Haginiwa S., Sadahiro T., Kojima H., Isomi M., Tamura F., Kurotsu S., Tani H., Muraoka N., Miyake N., Miyake K. (2019). Tbx6 induces cardiomyocyte proliferation in postnatal and adult mouse hearts. Biochem. Biophys. Res. Commun..

[76] Engel F.B., Schebesta M., Duong M.T., Lu G., Ren S., Madwed J.B., Jiang H., Wang Y., Keating M.T. (2005). p38 MAP kinase inhibition enables proliferation of adult mammalian cardiomyocytes. Genes Dev..

[77] Ponnusamy M., Liu F., Zhang Y.H., Li R.B., Zhai M., Liu F., Zhou L.Y., Liu C.Y., Yan K.W., Dong Y.H. (2019). Long Noncoding RNA CPR (Cardiomyocyte Proliferation Regulator) Regulates Cardiomyocyte Proliferation and Cardiac Repair. Circulation.

[78] Bersell K., Arab S., Haring B., Kuhn B. (2009). Neuregulin1/ErbB4 signaling induces cardiomyocyte proliferation and repair of heart injury. Cell.

[79] Kim A.R., Kim S.W., Lee B.W., Kim K.H., Kim W.H., Seok H., Lee J.H., Um J., Yim S.H., Ahn Y. (2020). Screening ginseng saponins in progenitor cells identifies 20(R)-ginsenoside Rh(2) as an enhancer of skeletal and cardiac muscle regeneration. Sci. Rep..

[80] Pianca N., Sacchi F., Umansky K.B., Chirivi M., Iommarini L., Da Pra S., Papa V., Bongiovanni C., Miano C., Pontis F. (2022). Glucocorticoid receptor antagonization propels endogenous cardiomyocyte proliferation and cardiac regeneration. Nat. Cardiovasc. Res..

[81] Harris B.N., Woo L.A., Perry R.N., Wallace A.M., Civelek M., Wolf M.J., Saucerman J.J. (2025). Dynamic map illuminates Hippo-cMyc module crosstalk driving cardiomyocyte proliferation. Development.

[82] Ahmed M.S., Nguyen N.U.N., Nakada Y., Hsu C.C., Farag A., Lam N.T., Wang P., Thet S., Menendez-Montes I., Elhelaly W.M. (2024). Identification of FDA-approved drugs that induce heart regeneration in mammals. Nat. Cardiovasc. Res..

[83] Yuan T., Wu M., Zhu C., Yu H., Pham M.D., Bottermann K., Mao Y., Wang Y., Langner M., Peitzsch M. (2025). Targeting miRNA-1a and miRNA-15b: A Novel Combinatorial Strategy to Drive Adult Cardiac Regeneration. Adv. Sci..

[84] Cheng Y.Y., Yan Y.T., Lundy D.J., Lo A.H., Wang Y.P., Ruan S.C., Lin P.J., Hsieh P.C. (2017). Reprogramming-derived gene cocktail increases cardiomyocyte proliferation for heart regeneration. EMBO Mol. Med..

[85] Eulalio A., Mano M., Dal Ferro M., Zentilin L., Sinagra G., Zacchigna S., Giacca M. (2012). Functional screening identifies miRNAs inducing cardiac regeneration. Nature.

[86] Renikunta H.V., Lazarow K., Gong Y., Shukla P.C., Nageswaran V., Giral H., Kratzer A., Opitz L., Engel F.B., Haghikia A. (2023). Large-scale microRNA functional high-throughput screening identifies miR-515-3p and miR-519e-3p as inducers of human cardiomyocyte proliferation. iScience.

[87] Magadum A., Renikunta H.V., Singh N., Estaras C., Kishore R., Engel F.B. (2022). Live cell screening identifies glycosides as enhancers of cardiomyocyte cell cycle activity. Front. Cardiovasc. Med..

[88] Titmarsh D.M., Glass N.R., Mills R.J., Hidalgo A., Wolvetang E.J., Porrello E.R., Hudson J.E., Cooper-White J.J. (2016). Induction of Human iPSC-Derived Cardiomyocyte Proliferation Revealed by Combinatorial Screening in High Density Microbioreactor Arrays. Sci. Rep..

[89] Meng F., Kwok M., Hui Y.C., Wei R., Hidalgo-Gonzalez A., Walentinsson A., Andersson H., Bjerre F.A., Wang Q.D., Andersen D.C. (2025). Matured hiPSC-derived cardiomyocytes possess dematuration plasticity. J. Mol. Cell. Cardiol. Plus.

[90] Sarma H., Upadhyaya M., Gogoi B., Phukan M., Kashyap P., Das B., Devi R., Sharma H.K. (2022). Cardiovascular Drugs: An Insight of In Silico Drug Design Tools. J. Pharm. Innov..

[91] Tabana Y., Babu D., Fahlman R., Siraki A.G., Barakat K. (2023). Target identification of small molecules: An overview of the current applications in drug discovery. BMC Biotechnol..

[92] Salmenov R., Mummery C., Ter Huurne M. (2024). Cell cycle visualization tools to study cardiomyocyte proliferation in real-time. Open Biol..

[93] Uosaki H., Andersen P., Shenje L.T., Fernandez L., Christiansen S.L., Kwon C. (2012). Direct contact with endoderm-like cells efficiently induces cardiac progenitors from mouse and human pluripotent stem cells. PLoS ONE.

[94] Engel F.B., Schebesta M., Keating M.T. (2006). Anillin localization defect in cardiomyocyte binucleation. J. Mol. Cell. Cardiol..

[95] Hesse M., Doengi M., Becker A., Kimura K., Voeltz N., Stein V., Fleischmann B.K. (2018). Midbody Positioning and Distance Between Daughter Nuclei Enable Unequivocal Identification of Cardiomyocyte Cell Division in Mice. Circ. Res..

[96] Kadow Z.A., Martin J.F. (2018). Distinguishing Cardiomyocyte Division From Binucleation. Circ. Res..

[97] Hao K., Nguyen T., Nakada Y., Walcott G., Wei Y., Wu Y., Garry D.J., Yao P., Zhang J. (2025). Newborn apical resection preserves the proliferative capacity of cardiomyocytes located throughout the left ventricle. Stem Cells.

[98] Leone M., Musa G., Engel F.B. (2018). Cardiomyocyte binucleation is associated with aberrant mitotic microtubule distribution, mislocalization of RhoA and IQGAP3, as well as defective actomyosin ring anchorage and cleavage furrow ingression. Cardiovasc. Res..

[99] Zebrowski D.C., Vergarajauregui S., Wu C.C., Piatkowski T., Becker R., Leone M., Hirth S., Ricciardi F., Falk N., Giessl A. (2015). Developmental alterations in centrosome integrity contribute to the post-mitotic state of mammalian cardiomyocytes. eLife.

[100] Ng D.C.H., Richards D.K., Mills R.J., Ho U.Y., Perks H.L., Tucker C.R., Voges H.K., Pagan J.K., Hudson J.E. (2020). Centrosome Reduction Promotes Terminal Differentiation of Human Cardiomyocytes. Stem Cell Rep..

[101] Chun Y.W., Miyamoto M., Williams C.H., Neitzel L.R., Silver-Isenstadt M., Cadar A.G., Fuller D.T., Fong D.C., Liu H., Lease R. (2023). Impaired Reorganization of Centrosome Structure Underlies Human Infantile Dilated Cardiomyopathy. Circulation.

[102] He L., Pu W., Liu X., Zhang Z., Han M., Li Y., Huang X., Han X., Li Y., Liu K. (2021). Proliferation tracing reveals regional hepatocyte generation in liver homeostasis and repair. Science.

[103] Liu X., Weng W., He L., Zhou B. (2023). Genetic recording of in vivo cell proliferation by ProTracer. Nat. Protoc..

[104] Liu X., Pu W., He L., Li Y., Zhao H., Li Y., Liu K., Huang X., Weng W., Wang Q.D. (2021). Cell proliferation fate mapping reveals regional cardiomyocyte cell-cycle activity in subendocardial muscle of left ventricle. Nat. Commun..

[105] Young A., Bradley L.A., Wolf M.J. (2022). In Vivo Methods to Monitor Cardiomyocyte Proliferation. J. Cardiovasc. Dev. Dis..

[106] Pilz P.M., Ward J.E., Chang W.T., Kiss A., Bateh E., Jha A., Fisch S., Podesser B.K., Liao R. (2022). Large and Small Animal Models of Heart Failure With Reduced Ejection Fraction. Circ. Res..

[107] De Villiers C., Riley P.R. (2020). Mouse models of myocardial infarction: Comparing permanent ligation and ischaemia-reperfusion. Dis. Models Mech..

[108] Gunata M., Parlakpinar H. (2021). A review of myocardial ischaemia/reperfusion injury: Pathophysiology, experimental models, biomarkers, genetics and pharmacological treatment. Cell Biochem. Funct..

[109] Zeymer U., Ludman P., Danchin N., Kala P., Laroche C., Sadeghi M., Caporale R., Shaheen S.M., Legutko J., Iakobsishvili Z. (2021). Reperfusion therapies and in-hospital outcomes for ST-elevation myocardial infarction in Europe: The ACVC-EAPCI EORP STEMI Registry of the European Society of Cardiology. Eur. Heart J..

[110] Gharacholou S.M., Alexander K.P., Chen A.Y., Wang T.Y., Melloni C., Gibler W.B., Pollack C.V., Ohman E.M., Peterson E.D., Roe M.T. (2010). Implications and reasons for the lack of use of reperfusion therapy in patients with ST-segment elevation myocardial infarction: Findings from the CRUSADE initiative. Am. Heart J..

[111] Thygesen K., Alpert J.S., Jaffe A.S., Chaitman B.R., Bax J.J., Morrow D.A., White H.D., Executive Group on behalf of the Joint European Society of Cardiology (ESC)/American College of Cardiology (ACC)/American Heart Association (AHA)/World Heart Federation (WHF) Task Force for the Universal Definition of Myocardial Infarction (2018). Fourth Universal Definition of Myocardial Infarction (2018). Circulation.

[112] Yu Y., Qin N., Lu X.A., Li J., Han X., Ni X., Ye L., Shen Z., Chen W., Zhao Z.A. (2019). Human embryonic stem cell-derived cardiomyocyte therapy in mouse permanent ischemia and ischemia-reperfusion models. Stem Cell Res. Ther..

[113] Xin M., Kim Y., Sutherland L.B., Murakami M., Qi X., McAnally J., Porrello E.R., Mahmoud A.I., Tan W., Shelton J.M. (2013). Hippo pathway effector Yap promotes cardiac regeneration. Proc. Natl. Acad. Sci. USA.

[114] Liu S., Li K., Wagner Florencio L., Tang L., Heallen T.R., Leach J.P., Wang Y., Grisanti F., Willerson J.T., Perin E.C. (2021). Gene therapy knockdown of Hippo signaling induces cardiomyocyte renewal in pigs after myocardial infarction. Sci. Transl. Med..

[115] Sun J., Wang L., Matthews R.C., Walcott G.P., Lu Y.A., Wei Y., Zhou Y., Zangi L., Zhang J. (2023). CCND2 Modified mRNA Activates Cell Cycle of Cardiomyocytes in Hearts With Myocardial Infarction in Mice and Pigs. Circ. Res..

[116] Gabisonia K., Prosdocimo G., Aquaro G.D., Carlucci L., Zentilin L., Secco I., Ali H., Braga L., Gorgodze N., Bernini F. (2019). MicroRNA therapy stimulates uncontrolled cardiac repair after myocardial infarction in pigs. Nature.

[117] Raso A., Dirkx E., Sampaio-Pinto V., El Azzouzi H., Cubero R.J., Sorensen D.W., Ottaviani L., Olieslagers S., Huibers M.M., de Weger R. (2021). A microRNA program regulates the balance between cardiomyocyte hyperplasia and hypertrophy and stimulates cardiac regeneration. Nat. Commun..

[118] Yu Z., Zhang S., Bogomolovas J., Chen J., Evans S.M. (2025). Intronic RNAscope probes enable precise identification of cardiomyocyte nuclei and cell cycle activity. Commun. Biol..

[119] Sun Y., Jiang C., Hong H., Liu J., Qiu L., Huang Y., Ye L. (2019). Effects of hypoxia on cardiomyocyte proliferation and association with stage of development. Biomed. Pharmacother..

[120] Calcagno D.M., Taghdiri N., Ninh V.K., Mesfin J.M., Toomu A., Sehgal R., Lee J., Liang Y., Duran J.M., Adler E. (2022). Single-cell and spatial transcriptomics of the infarcted heart define the dynamic onset of the border zone in response to mechanical destabilization. Nat. Cardiovasc. Res..

[121] Liu N., Pei J., Xie Y., Xuan H., Jiang N., Wang J., Gao Y., Li Y., Li X., Liu W. (2025). PTMA controls cardiomyocyte proliferation and cardiac repair by enhancing STAT3 acetylation. Sci. Adv..

[122] Cui M., Wang Z., Chen K., Shah A.M., Tan W., Duan L., Sanchez-Ortiz E., Li H., Xu L., Liu N. (2020). Dynamic Transcriptional Responses to Injury of Regenerative and Non-regenerative Cardiomyocytes Revealed by Single-Nucleus RNA Sequencing. Dev. Cell.

[123] Wang H., Dong Y., Song Y., Colon M., Yapundich N., Ricketts S., Liu X., Farber G., Qian Y., Qian L. (2025). Charting Postnatal Heart Development Using In Vivo Single-Cell Functional Genomics. bioRxiv.

[124] Nakada Y., Zhou Y., Gong W., Zhang E.Y., Skie E., Nguyen T., Wei Y., Zhao M., Chen W., Sun J. (2022). Single Nucleus Transcriptomics: Apical Resection in Newborn Pigs Extends the Time Window of Cardiomyocyte Proliferation and Myocardial Regeneration. Circulation.

[125] Copeland K.M., Brazile B.L., Butler J.R., Cooley J., Brinkman-Ferguson E., Claude A., Lin S., Rais-Rohani S., Welch B., McMahan S.R. (2022). Investigating the Transient Regenerative Potential of Cardiac Muscle Using a Neonatal Pig Partial Apical Resection Model. Bioengineering.

[126] Ellman D.G., Slaiman I.M., Mathiesen S.B., Andersen K.S., Hofmeister W., Ober E.A., Andersen D.C. (2021). Apex Resection in Zebrafish (Danio rerio) as a Model of Heart Regeneration: A Video-Assisted Guide. Int. J. Mol. Sci..

[127] Galow A.M., Wolfien M., Muller P., Bartsch M., Brunner R.M., Hoeflich A., Wolkenhauer O., David R., Goldammer T. (2020). Integrative Cluster Analysis of Whole Hearts Reveals Proliferative Cardiomyocytes in Adult Mice. Cells.

[128] Zhang Y., Gago-Lopez N., Li N., Zhang Z., Alver N., Liu Y., Martinson A.M., Mehri A., MacLellan W.R. (2019). Single-cell imaging and transcriptomic analyses of endogenous cardiomyocyte dedifferentiation and cycling. Cell Discov..

[129] Koenig A.L., Shchukina I., Amrute J., Andhey P.S., Zaitsev K., Lai L., Bajpai G., Bredemeyer A., Smith G., Jones C. (2022). Single-cell transcriptomics reveals cell-type-specific diversification in human heart failure. Nat. Cardiovasc. Res..

[130] Morikawa Y., Kim J.H., Li R.G., Liu L., Liu S., Deshmukh V., Hill M.C., Martin J.F. (2025). YAP Overcomes Mechanical Barriers to Induce Mitotic Rounding and Adult Cardiomyocyte Division. Circulation.

[131] Bouwman M., de Bakker D.E.M., Honkoop H., Giovou A.E., Versteeg D., Boender A.R., Nguyen P.D., Slotboom M., Colquhoun D., Vigil-Garcia M. (2025). Cross-species comparison reveals that Hmga1 reduces H3K27me3 levels to promote cardiomyocyte proliferation and cardiac regeneration. Nat. Cardiovasc. Res..

[132] Leach J.P., Heallen T., Zhang M., Rahmani M., Morikawa Y., Hill M.C., Segura A., Willerson J.T., Martin J.F. (2017). Hippo pathway deficiency reverses systolic heart failure after infarction. Nature.

[133] Xiang F.L., Guo M., Yutzey K.E. (2016). Overexpression of Tbx20 in Adult Cardiomyocytes Promotes Proliferation and Improves Cardiac Function After Myocardial Infarction. Circulation.

[134] Lin Z., von Gise A., Zhou P., Gu F., Ma Q., Jiang J., Yau A.L., Buck J.N., Gouin K.A., van Gorp P.R. (2014). Cardiac-specific YAP activation improves cardiac function and survival in an experimental murine MI model. Circ. Res..

[135] Tan Y., Duan X., Wang B., Liu X., Zhan Z. (2023). Murine neonatal cardiac B cells promote cardiomyocyte proliferation and heart regeneration. npj Regen. Med..

[136] Li J., Yang K.Y., Tam R.C.Y., Chan V.W., Lan H.Y., Hori S., Zhou B., Lui K.O. (2019). Regulatory T-cells regulate neonatal heart regeneration by potentiating cardiomyocyte proliferation in a paracrine manner. Theranostics.

[137] Wang Z., Cui M., Shah A.M., Tan W., Liu N., Bassel-Duby R., Olson E.N. (2020). Cell-Type-Specific Gene Regulatory Networks Underlying Murine Neonatal Heart Regeneration at Single-Cell Resolution. Cell Rep..

[138] Wei K.H., Lin I.T., Chowdhury K., Lim K.L., Liu K.T., Ko T.M., Chang Y.M., Yang K.C., Lai S.L. (2023). Comparative single-cell profiling reveals distinct cardiac resident macrophages essential for zebrafish heart regeneration. eLife.

[139] Constanty F., Wu B., Wei K.H., Lin I.T., Dallmann J., Guenther S., Lautenschlaeger T., Priya R., Lai S.L., Stainier D.Y.R. (2025). Border-zone cardiomyocytes and macrophages regulate extracellular matrix remodeling to promote cardiomyocyte protrusion during cardiac regeneration. Nat. Commun..

[140] Feng J., Li Y., Li Y., Yin Q., Li H., Li J., Zhou B., Meng J., Lian H., Wu M. (2024). Versican Promotes Cardiomyocyte Proliferation and Cardiac Repair. Circulation.

[141] Wang Y., Yao F., Wang L., Li Z., Ren Z., Li D., Zhang M., Han L., Wang S.Q., Zhou B. (2020). Single-cell analysis of murine fibroblasts identifies neonatal to adult switching that regulates cardiomyocyte maturation. Nat. Commun..

[142] Smits A.M., Dronkers E., Goumans M.J. (2018). The epicardium as a source of multipotent adult cardiac progenitor cells: Their origin, role and fate. Pharmacol. Res..

[143] Eroglu E., Yen C.Y.T., Tsoi Y.L., Witman N., Elewa A., Joven Araus A., Wang H., Szattler T., Umeano C.H., Sohlmer J. (2022). Epicardium-derived cells organize through tight junctions to replenish cardiac muscle in salamanders. Nat. Cell Biol..

[144] Zhou B., Honor L.B., Ma Q., Oh J.H., Lin R.Z., Melero-Martin J.M., von Gise A., Zhou P., Hu T., He L. (2012). Thymosin beta 4 treatment after myocardial infarction does not reprogram epicardial cells into cardiomyocytes. J. Mol. Cell Cardiol..

[145] Sun J., Peterson E.A., Wang A.Z., Ou J., Smith K.E., Poss K.D., Wang J. (2022). hapln1 Defines an Epicardial Cell Subpopulation Required for Cardiomyocyte Expansion During Heart Morphogenesis and Regeneration. Circulation.

[146] Chen X., Zhong X., Huang G.N. (2024). Heart regeneration from the whole-organism perspective to single-cell resolution. npj Regen. Med..

[147] Dong Y., Yang Y., Wang H., Feng D., Nist E., Yapundich N., Spurlock B., Craft M., Qian L., Liu J. (2024). Single-cell chromatin profiling reveals genetic programs activating proregenerative states in nonmyocyte cells. Sci. Adv..

[148] Wang H., Yang Y., Qian Y., Liu J., Qian L. (2022). Delineating chromatin accessibility re-patterning at single cell level during early stage of direct cardiac reprogramming. J. Mol. Cell. Cardiol..

[149] Sahoo S., Kariya T., Ishikawa K. (2021). Targeted delivery of therapeutic agents to the heart. Nat. Rev. Cardiol..

[150] Vekstein A.M., Wendell D.C., DeLuca S., Yan R., Chen Y., Bishawi M., Devlin G.W., Asokan A., Poss K.D., Bowles D.E. (2022). Targeted Delivery for Cardiac Regeneration: Comparison of Intra-coronary Infusion and Intra-myocardial Injection in Porcine Hearts. Front. Cardiovasc. Med..

[151] Feng W., He P., Wang Z., Li W. (2025). Breakthroughs of ultrasound-targeted microbubble destruction in treating myocardial ischemia-reperfusion injury: From angiogenesis regulation to precise inflammation suppression. Drug Deliv..

[152] Mc Cafferty S., De Temmerman J., Kitada T., Becraft J.R., Weiss R., Irvine D.J., Devreese M., De Baere S., Combes F., Sanders N.N. (2021). In Vivo Validation of a Reversible Small Molecule-Based Switch for Synthetic Self-Amplifying mRNA Regulation. Mol. Ther..

[153] Karimi M., Ghasemi A., Sahandi Zangabad P., Rahighi R., Moosavi Basri S.M., Mirshekari H., Amiri M., Shafaei Pishabad Z., Aslani A., Bozorgomid M. (2016). Smart micro/nanoparticles in stimulus-responsive drug/gene delivery systems. Chem. Soc. Rev..

[154] Toischer K., Zhu W., Hunlich M., Mohamed B.A., Khadjeh S., Reuter S.P., Schafer K., Ramanujam D., Engelhardt S., Field L.J. (2017). Cardiomyocyte proliferation prevents failure in pressure overload but not volume overload. J. Clin. Investig..

[155] Heallen T., Zhang M., Wang J., Bonilla-Claudio M., Klysik E., Johnson R.L., Martin J.F. (2011). Hippo pathway inhibits Wnt signaling to restrain cardiomyocyte proliferation and heart size. Science.

[156] Wintruba K.L., Wolf M.J., van Berlo J.H., Saucerman J.J. (2025). Advances in Drug Discovery for Cardiomyocyte Proliferation. Curr. Treat. Options Cardiovasc. Med..

[157] Bois A., Grandela C., Gallant J., Mummery C., Menasche P. (2025). Revitalizing the heart: Strategies and tools for cardiomyocyte regeneration post-myocardial infarction. npj Regen. Med..

[158] Burjanadze G., Gorgodze N., Aquaro G.D., Gabisonia K., Carlucci L., Pachauri M., Turreni F., Secco I., Bernini F., Zentilin L. (2025). Delayed miR-199a Administration After Myocardial Infarction Precludes Pro-Regenerative Effects. JACC Basic Transl. Sci..

[159] Fan C., Oduk Y., Zhao M., Lou X., Tang Y., Pretorius D., Valarmathi M.T., Walcott G.P., Yang J., Menasche P. (2020). Myocardial protection by nanomaterials formulated with CHIR99021 and FGF1. JCI Insight.

[160] Liu H., Zhang C.H., Ammanamanchi N., Suresh S., Lewarchik C., Rao K., Uys G.M., Han L., Abrial M., Yimlamai D. (2019). Control of cytokinesis by beta-adrenergic receptors indicates an approach for regulating cardiomyocyte endowment. Sci. Transl. Med..

[161] El Khoudary S.R., Fabio A., Yester J.W., Steinhauser M.L., Christopher A.B., Gyngard F., Adams P.S., Morell V.O., Viegas M., Da Silva J.P. (2021). Design and rationale of a clinical trial to increase cardiomyocyte division in infants with tetralogy of Fallot. Int. J. Cardiol..

